# Data quality and timeliness analysis for post-vaccination adverse event cases reported through healthcare data exchange to FDA BEST pilot platform

**DOI:** 10.3389/fpubh.2024.1379973

**Published:** 2024-07-08

**Authors:** Matthew Deady, Ray Duncan, Lance D. Jones, Arianna Sang, Brian Goodness, Abhishek Pandey, Sylvia Cho, Richard A. Forshee, Steven A. Anderson, Hussein Ezzeldin

**Affiliations:** ^1^IBM Consulting, Washington, DC, United States; ^2^Department of Enterprise Information Services and Pediatrics, Los Angeles, Cedars-Sinai Health System, CA, United States; ^3^Center for Biologics Evaluation and Research, United States Food and Drug Administration, Silver Spring, MD, United States

**Keywords:** data quality, fast healthcare interoperability resources (FHIR), interoperability, real-world data (RWD), electronic health record (EHR), public health, adverse event (AE), health information technology

## Abstract

**Introduction:**

This study is part of the U.S. Food and Drug Administration (FDA)’s Biologics Effectiveness and Safety (BEST) initiative, which aims to improve the FDA’s postmarket surveillance capabilities by using real-world data (RWD). In the United States, using RWD for postmarket surveillance has been hindered by the inability to exchange clinical data between healthcare providers and public health organizations in an interoperable format. However, the Office of the National Coordinator for Health Information Technology (ONC) has recently enacted regulation requiring all healthcare providers to support seamless access, exchange, and use of electronic health information through the interoperable HL7 Fast Healthcare Interoperability Resources (FHIR) standard. To leverage the recent ONC changes, BEST designed a pilot platform to query and receive the clinical information necessary to analyze suspected AEs. This study assessed the feasibility of using the RWD received through the data exchange of FHIR resources to study post-vaccination AE cases by evaluating the data volume, query response time, and data quality.

**Materials and methods:**

The study used RWD from 283 post-vaccination AE cases, which were received through the platform. We used descriptive statistics to report results and apply 322 data quality tests based on a data quality framework for EHR.

**Results:**

The volume analysis indicated the average clinical resources for a post-vaccination AE case was 983.9 for the median partner. The query response time analysis indicated that cases could be received by the platform at a median of 3 min and 30 s. The quality analysis indicated that most of the data elements and conformance requirements useful for postmarket surveillance were met.

**Discussion:**

This study describes the platform’s data volume, data query response time, and data quality results from the queried postvaccination adverse event cases and identified updates to current standards to close data quality gaps.

## 1 Introduction

The U.S. Food and Drug Administration (FDA) Center for Biologics Evaluation and Research (CBER) is responsible for ensuring the safety, purity, potency, and effectiveness of biological products. This includes vaccines, allergenics, blood and blood products, cells, tissues, and gene therapies for the prevention, diagnosis, and treatment of human diseases, conditions, or injuries (1). While FDA-approved biologics are comprehensively assessed for safety concerns during early and pivotal trials, postmarket surveillance systems that capture larger patient populations could power studies to detect rare adverse events (AEs). These improvements are particularly important to help detect AEs that require medical attention allowing the FDA and its reporting partner, Centers for Disease Control and Prevention (CDC), to offer guidance about severe or potentially life-threatening events.

Currently, vaccine surveillance is primarily carried out by the Vaccine Adverse Event Reporting System (VAERS), jointly administered by the FDA and CDC. VAERS accepts spontaneous reports of suspected vaccine AEs after administration of any vaccine licensed or authorized for emergency use in the United States, and continues to be a valuable early warning system to identify rare AEs (2) following clinical trials for postmarket surveillance. VAERS, like all passive surveillance in any setting, has limitations, including but not limited to, issues with underreporting (3), reporting quality (4), and obtaining the data necessary to detect a causal relationship between a vaccination and an adverse event (5).

Reports to VAERS are often incomplete and lack sufficient information to inform regulatory decision-making. To obtain the information necessary to follow up on a case, the FDA’s VAERS team must contact the reporting physician or their organization directly, which involves requesting and exchanging data through a manual process involving both the FDA and the reporting healthcare organization (6). This process can be time-consuming and lead to inconsistencies in data format, quality, volume, and lead-time for data received by the FDA from different organizations. To avoid delays in these investigations, FDA and CDC may incorporate analyses from other countries as a basis to issue United States guidance (7). For example, Israel’s retrospective analysis of data from a large health care provider that identified a probable link between the second dose of the Pfizer COVID-19 vaccine and myocarditis cases was reviewed as part of the evidence leading to an updated Fact Sheet for Healthcare Providers Administering Vaccine (8).

To improve its post-marketing surveillance capabilities, CBER established an active surveillance system, the Biologics Effectiveness and Safety (BEST) Initiative in 2017, to build data assets, analytics, and infrastructure for a large-scale, efficient, postmarket active surveillance system with the ability to evaluate the safety and effectiveness of biologic products (9). The BEST system is a collection of real-world data (RWD) sources, which are information related to patient health status and/or the delivery of health care routinely collected from several sources, which may include electronic health records (EHRs) and claims data (10). In particular, EHR databases are a rich source of information that may help address the limitations of VAERS. They include entire populations of patients, which can help identify any underreported cases and allow for detailed investigations of individual patient cases.

Biologics Effectiveness and Safety, in its initial stage, focused on negotiating agreements with data partners to share their EHR data. These BEST partnerships allow for epidemiologic studies based on the RWD of the partner’s patient populations (11–13). However, because of the difficulties in negotiating these agreements, BEST was able to obtain EHR databases from three partners only. Because of the limited number of partners, there is a potential for undercounting of AEs and a lack of ability to easily obtain additional data for reported cases from providers outside these partners. Increasing the capabilities of the BEST system to address the limitations of VAERS is paramount for public health efforts where rapidly evaluating biologic products is a necessity. The current limitations were highlighted by the COVID-19 pandemic, as the Emergency Use Authorization (EUA) of three novel COVID-19 vaccines (Pfizer-BioNTech, Moderna, and Novavax) became pertinent to the FDA (5).

For these reasons, BEST staff are evaluating the use of a healthcare information exchange (HIE)-based platform for improved postmarket vaccine active surveillance that could receive automated AE reports and query-reported VAERS cases to generate additional EHR data as necessary. This BEST pilot platform aims to allow the FDA to address underreporting of AEs by making the AE reporting process for healthcare providers digital and automatic. It also aims to allow the FDA to receive richer clinical data sets for its active surveillance system, including unstructured clinician notes, often critical for postmarket analysis, without requiring further translation to a common data model by one or both parties. This is expected to enable the BEST pilot platform to receive automated AE reports and query EHR data depends on the ability to collect and exchange clinical EHR RWD without losing critical data, such as vaccine administration and other information required for VAERS submission, including detail not found in claims data, such as clinical notes.

The platform hopes to take advantage of the Office of the National Coordinator (ONC) Cures Act Final Rule, which supports seamless and secure access, exchange, and use of Electronic Health Information (EHI) by requiring certified EHR to support Fast Healthcare Interoperability Resources (FHIR) as the federally-mandated standard (14). The FHIR standard defines a set of basic building blocks, the so-called resources, which are a generic definition of common health care concepts (e.g., patient, observation, practitioner, device, and condition) (15). FHIR was developed by Health Level Seven, Inc. (HL7) (15) to facilitate interoperability between health systems (16), and was designed to improve existing standards by reducing implementation complexity without losing information integrity (15, 17). Seamless access is achieved by requiring data to be interoperable, which is defined as two or more software or systems with the ability to read and make use of the information received from one another. To better define the standard and its implementation, the ONC for Health Information Technology released a set of minimum requirements known as the United States Core Data for Interoperability (USCDI) (18). The Argonaut Project FHIR accelerator group has published the U.S. Core Implementation Guide (IG) that describes how to exchange USCDI data using FHIR (19). As of December 31, 2022, the ONC enacted the requirement that EHR vendors must provide FHIR application programming interfaces (APIs) supporting the first versions of the USCDI (v1) and U.S. Core IG (v3.1.1) to all customers or be at risk of losing their Healthcare IT developer certification (20).

Previously, without these requirements, it was difficult to set up a RWD study using EHR data combined from several different health providers given the issues with consistency, standardization, and secure transfer across partner data sets. Public health organizations often perform surveillance studies across several EHR datasets stored and governed by separate data use agreements, like BEST’s initial network of negotiated agreements with healthcare providers described above. This study is an initial attempt to assess the impact of ONC’s new requirements on creating a feasible technical alternative for public health entities to access a large set of EHR data across many healthcare provider partners and, additionally, to identify any new challenges with this approach. To limit the scope of our study, we focused our analysis on the feasibility of using this method for the FDA’s vaccine safety postmarket surveillance tasks. However, the findings should be generally applicable to any public health organization interested in using this technology to improve their collection of EHR data. The questions for this study include:

1. Are the data elements sufficiently populated to inform FDA’s vaccine postmarket surveillance activities? We assessed the completeness, conformance, and plausibility of data elements that map to the FDA VAERS form or were identified as being helpful for clinical review of a postvaccination adverse event by clinicians. We then researched whether these elements were included in current or future versions of the USCDI/U.S. Core requirements to evaluate how well the current state of data partners’ FHIR resources meet these requirements or may be expected to improve with future USCDI/U.S. Core IG.
2. What is the quantity and type of data available? Understanding the quantity of data provided for a patient case helps to answer questions about the clinical robustness of the data and technical questions around how to design a system with correct storage and data pipeline functionality.
3. How fast can the data be received through a HIE using FHIR, such as the BEST pilot platform? The speed that the data can be transferred from the healthcare provider data partners to the platform will allow FDA to understand how much this new technology can decrease the time to receipt of data and also inform how to design a system with an efficient workflow.
4. What level of effort should be expected to onboard a new partner to the exchange platform and what are the expected challenges or difficulties? Inability to easily and quickly add a new data partner to the system limits the future potential to scale the system to a nationwide, active surveillance system.

This study attempts to answer these questions through the process of onboarding 11 data partners and then completing data volume, data query response time, and data quality assessments. Any gaps identified by the data quality analysis can help inform the FDA and the healthcare informaticist community about the current state of FHIR data exchange for a public health use case, given the way in which current and future, planned versions of the USCDI and/or U.S. Core IG requirements are being implemented. These analyses are also expected to provide recommendations for changes in future versions of the USCDI/U.S. Core requirements so that the RWD can be made readily available to public health authorities. BEST staff will use outcomes from these analyses to inform the initiative’s long-term vision of scaling the platform to a nationwide, active surveillance system.

## 2 Materials and methods

### 2.1 Study period

The study period covered the timeframe starting with the first administration of COVID-19 vaccinations, following their Emergency Use Authorization (EUA), dated December 14, 2020, until the cut-off date for partners to submit their adverse event cases by May 1, 2023. Each of the 11 healthcare exchange partners identified a set of 30 or more unique post-vaccination patient AE cases within this study period to send to BEST’s exchange platform for assessment of the received data. We requested that our data partners identify, if available, the dates of the immunization and encounter related to the selected AE case, which would serve as start and end dates for the case. If received, these dates allowed the team to limit the FHIR data queried for several FHIR resources to only include events within the start and end dates for a case. We will refer to this as the “limited case window.” The limited case window began with the immunization date and continued for 10 days after the end of the encounter for the adverse event. If that date was not provided, the start of the adverse event encounter was used. The “limited case window” was about a month for most cases where we received the immunization or AE dates from the partner. However, five of our partners were not able to provide those dates. For the cases provided by these partners, we received the “entire study period” (December 14, 2020, to May 1, 2023) for each resource. The limited case window both limited the amount of data being accessed (following the principal of minimal use for health records) and limited the amount of data being transmitted and processed.

### 2.2 Data

Data for the AE cases were collected from 11 healthcare organizations connected to eHealth Exchange (eHX) that volunteered to participate in the pilot. eHX is a Health Information Exchange (HIE) that covers 77% of all United States hospitals, 61 state and regional health information exchanges, and five federal agencies. It uses a common set of standards and specifications to establish a trusted, interoperable connection to securely share detailed clinical information (21). Our platform was able to take advantage of the pre-negotiated, common agreement, Data Use and Reciprocal Support Agreement (DURSA) that all providers using the exchange must sign to participate. This allowed our platform timelier access to EHR RWD as opposed to negotiating data use with each partner, individually, using separate legal agreements. A partner that signed the DURSA would simply have to opt into the public health use case to allow the BEST pilot platform to receive RWD for post-vaccination adverse event cases from that partner through the exchange. We recruited the healthcare organizations that opted in for this study from among the existing eHX partners. Several incentives were offered to enhance participation, including eHX fee reimbursements, clarification of the purpose of public health use, and the possibility of research collaboration. All healthcare organizations that participated in this pilot used Epic EHR software to store and send their patient healthcare data. Table 1 characterizes the healthcare organizations that agreed to participate in the study. In Table 2, we captured the count of partners using each Epic release since different releases may affect the volume, query response time, or quality of the data received.

**Table 1:** Data partnerships onboarding characteristics.

Metric	Size (millions of patients)
Min	0.2
Max	3.9
Median	2.5
Average	1.9
Total	21.3

**Table 2:** Data partners epic version at study time.

Metric	Count of partners
May 2022	2
November 2022	7
February 2023	2
Total	11

**Figure 1:**
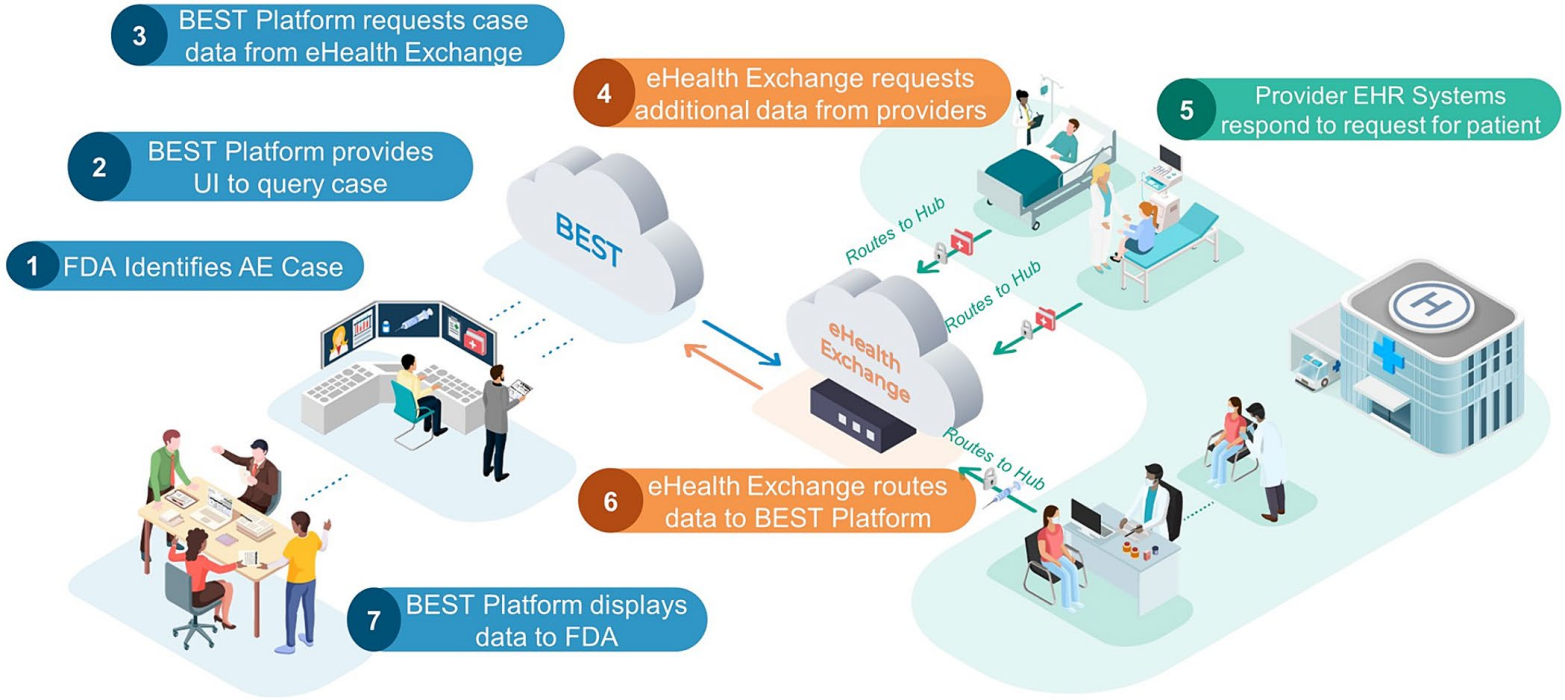
FDA BEST pilot platform. This FDA Biologics Effectiveness and Safety (BEST) Pilot Platform allows an FDA reviewer to query for additional clinical information for a patient with a probable AE. Listed below are the steps of the process: (1) FDA identifies AE case: an FDA reviewer, using VAERS adverse event case report, identify case of interest that requires additional data. (2) BEST Platform provides UI to query case: the FDA reviewer uses patient demographics provided in the VAERS report to send request for additional data. (3) BEST Platform request case data from eHealth Exchange. (4) eHealth Exchange requests additional data from providers: eHealth Exchange requests additional data for patient case from all participating health provider partners. (5) Provider EHR systems respond to request for patient: all health provider partners check their EHR databases for patients that match the queried demographics. If there is a match, the provider will send a FHIR bundle of agreed upon resource types for the patient case to eHealth exchange. (6) eHealth Exchange routes data to BEST Platform. (7) BEST Platform displays data to FDA: data are displayed in a custom chart review application. The left-hand side (blue) components are representative of the contractor-managed FDA BEST Platform, which includes several BEST-developed applications and services. This section also includes review of data by the FDA. The center (orange) cloud is representative of the eHealth Exchange, which serves as an intermediary between the FDA BEST Platform and any providers exchanging data. The right-hand side (green) components are representative of providers that are members of the eHealth Exchange. These are the sites where vaccinations/other biologic treatments occur and where any adverse event data would be queried for and sent back to FDA. Dotted arrows represent data flowing internally to one of the three systems described above while the solid arrows represent communication between systems. Adapted from “Validation of a Computable Phenotype for Myocarditis/Pericarditis Following COVID-19 Vaccinations Using a Pilot Active Surveillance Electronic Healthcare Data Exchange Platform” JMIR Preprints.

The healthcare exchange partners provided BEST with the demographic information needed to retrieve clinical data for the identified set of 30 or more unique post-vaccination patient AE cases discussed in the Study Period section. The query was sent by the BEST pilot platform to eHX, which in turn was able to query the data partner’s Epic FHIR APIs. The obtained clinical data were then sent back to the platform for evaluation. Figure 1 demonstrates how data were queried and exchanged through the system. Given the study timing, the FHIR data received were expected to match the currently required USCDI (v1) and U.S. Core IG (3.1.1) requirements. For the post-vaccination AE case selection, data partners were instructed to prioritize post COVID-19 vaccination cases in addition to other cases occurring within the study period. In cases where a data partner had less than 30 post-COVID-19 vaccination cases available, post-vaccination cases from other vaccinations, or those occurring slightly before the study period, were accepted.

As shown in Table 3, DiagnosticReport, DocumentReference, and all Observations were filtered by date for this “limited case window.” Both “limited case window” and “entire study period” cases received all the resource types that did not need to be limited by temporal windows, including those that are useful in reviewing a patient’s history (e.g., Conditions, Immunizations), in identifying another case range to pull (Encounters), were unable to be filtered (MedicationRequest), or that had a small volume of resources (AllergyIntolerance, Procedure). Lastly, both types of cases received resources referenced by another resource received (e.g., the location of an encounter).

**Table 3:** FHIR Resources Received and Analyzed.

Resource	Date period
AllergyIntolerance	Full clinical history
Condition	Full clinical history
DiagnosticReport	Limited case window/Entire study period (12/14/2020–05/01/2023)
DocumentReference	Limited case window/Entire study period (12/14/2020–05/01/2023)
Encounter	Full clinical history
Immunization	Full clinical history
Location	All referenced resources
Medication	All referenced resources
MedicationRequest	Full clinical history
Observation	Limited case window/Entire study period (12/14/2020–05/01/2023)
Patient	Single patient resource
Practitioner	All referenced resources
Procedure	Full clinical history

FHIR, Fast healthcare interoperability resources.

Occasionally, we were unable to pull data for a patient case using the demographics provided by our data partner. This occurred for a variety of reasons. In some cases, the demographic information provided matched more than one patient. In this case, eHX would not receive data for any patient that matched, given the possibility that one or more of the cases may not be the patient AE cases queried and thus the public health use case justification for data retrieval would not apply. In other cases, patients may have “Break-the-Glass” protection. This is when an additional layer of access controls has been instituted that require an additional “Break-the-Glass” security procedure for sensitive patients, such as employees or celebrities (22, 23). If a case was unable to be pulled into the BEST pilot platform because of these or other errors, we continued to query other AE cases from that partner until we had 30 cases from that partner or we had exhausted all of the cases provided by the partner. If the platform was unable to receive data for the full 30 cases for a partner, the analysis was done on the cases that could be received by the platform. In total, 283 patient cases were obtained.

### 2.3 Data volume analysis

To understand the volume of clinical data per patient case, we performed an analysis calculating the average number of semantically-relevant clinical events for all 283 patient cases received by the partner and by the event type. Semantically relevant clinical data excludes any FHIR resources with status values such as “entered in error” or “not done,” indicating the action never occurred. The list of statuses removed is included in Appendix Table 1 in the Multimedia appendix. These resources are often missing key data by design given the absence of a clinical action. To measure the number and type of clinical events, we used the count of FHIR resources per patient by resource type. FHIR resources are modular components that make up the basic data exchange and format of the FHIR data. Resources contain a collection of data elements based on the type (e.g., patient, encounter, and condition) and references to other resources. For volume calculations, we did not include resources referenced by any other resource (e.g., practitioner resources referenced by an encounter, medication resources referenced by a medication request) because they are repeatedly referenced across resources both within and across patients, which would artificially inflate volume through double counting. To compare variation among partner cases, we calculated the minimum, maximum, median, and average of the average resource counts for the 11 partners by resource type (i.e., minimum for observation resource type would be calculated as the average observation per patient at the partner with the lowest average observation per patient). Lastly, we randomly sampled 100 semantically-relevant resources for each resource type from the entire patient case population to calculate the average resource size in bytes per resource. The results for these measures are divided into two tables, one for all “limited case window” (Table 4) cases and the other for all “entire study period” cases (Table 5).

**Table 4:** Average and range of values for limited case window partner’s average per patient resources per patient by resource type and estimated average size in bytes.

Resource	Median	Average (Min, Max)	Est. Size/Resource (KB)	Est. avg. total size per case (Min, Max) (KB)
Allergy intolerance	2.8	2.7 (1.2, 3.7)	3.18	8.59 (3.82, 11.77)
Condition—Encounter diagnosis	239.1	205.9 (40.6, 321.3)	3.08	634.17 (125.05, 989.60)
Condition—Problems/Health concerns	16.9	14.6 (4.8, 23.6)	3.05	44.59 (14.64, 71.98)
Diagnostic report	20.0	27.4 (16.3, 63.2)	7.05	193.12 (114.92, 445.56)
Document reference—Clinical notes	60.2	67.7 (30.3, 124.2)	46.08	3119.62 (1396.22, 5723.14)
Document reference—External CDA	51.8	60.1 (22.6, 118.6)	22.53	1353.93 (509.18, 2672.06)
Document reference—correspondence	18.8	20.2 (2.3, 39.1)	118.78	2399.44 (273.19, 4.644.30)
Document reference—Imaging result	22.7	21.5 (3.9, 36.9)	108.54	2333.70 (423.31, 4005.13)
Document reference—Handoff	0.0	0.3 (0.0, 1.5)	33.79	10.14 (0.00, 50.69)
Encounter	218.2	206.1 (54.4, 355.8)	4.22	869.33 (229.57, 1501.48)
Immunization	14.9	14.8 (3.6, 23.7)	2.29	33.89 (8.24, 54.27)
Medication request	114.2	131.3 (19.5, 265.5)	4.84	635.89 (94.38, 1285.02)
Observation—Lab test	89.2	130.1 (71.0, 343.0)	3.65	475.20 (259.15, 1253.05)
Observation—Vital sign	51.8	49.4 (13.0, 84.9)	3.28	162.13 (42.64, 278.47)
Observation—LDA	3.6	8.3 (0.0, 25.4)	3.98	33.06 (0.00, 101.09)
Observation—Social history	7.0	8.6 (5.5, 18.2)	2.70	23.22 (14.85, 49.14)
Observation—Other	-	-	3.18	-
Procedure—Order	7.6	9.9 (1.5, 27.7)	3.08	32.01 (4.62, 85.32)
Procedure—Surgical history	5.1	5.1 (2.2, 8.4)	3.05	39.71 (6.71, 25.62)
Total resources	1062.3	983.9 (393.9, 1510.6)	12.61	12402.72 (4967.08, 19048.67)

CDA, Clinical document architecture; LDA, Lines drains and airways.

**Table 5:** Average and range of values for entire study period partner’s average per patient resources per patient by resource type and estimated average size in bytes.

Resource	Median	Average (Min, Max)	Est. Size/Resource (KB)	Est. Avg. Total Size per Case (Min, Max) (KB)
Allergy intolerance	4.5	4.1 (1.8, 6.3)	3.18	13.05 (5.72, 20.03)
Condition—Encounter diagnosis	120.0	181.3 (81.0, 330.5)	3.08	558.40 (249.48, 1017.94)
Condition—Problems/Health concerns	12.2	15.2 (7.4, 25.5)	3.05	46.42 (22.57, 77.78)
Diagnostic report	42.3	50.0 (24.0, 83.6)	7.05	352.40 (169.20, 589.38)
Document reference—Clinical notes	184.6	204.9 (33.7, 398.9)	46.08	9441.79 (1522.90, 18381.31)
Document reference—External CDA	-	42.5 (0.0, 164.1)	22.53	957.44 (0.00, 3697.17)
Document reference—Correspondence	14.4	22.2 (6.3, 49.9)	118.78	2637.00 (748.31, 5927.12)
Document reference—Imaging result	22.0	33.0 (13.1, 83.4)	108.54	3581.95 (1421.87, 9062.24)
Document reference—Handoff	-	0.2 (0.0, 0.8)	33.79	6.76 (0.00, 27.03)
Encounter	122.1	170.5 (88.3, 288.7)	4.22	719.17 (372.63, 1218.31)
Immunization	10.0	9.3 (5.0, 14.1)	2.29	21.30 (11.45, 32.29)
Medication request	103.2	206.3 (31.5, 624,2)	4.84	999.11 (152.46, 3021.13)
Observation—Lab test	279.0	348.4 (76.9, 834.3)	3.65	1272.56 (280.69, 3045.20)
Observation—Vital Sign	114.8	178.4 (16.9, 454.1)	3.28	585.51 (55.43, 1489.45)
Observation—LDA	12.4	23.3 (0.1, 84.1)	3.98	92.80 (0.40, 334.72)
Observation—Social history	10.0	13.8 (5.8, 32.4)	2.70	37.26 (15.66, 87.48)
Observation—Other	-	-	3.18	-
Procedure—Order	4.8	5.2 (2.1, 8.0)	3.08	16.81 (6.47, 24.64)
Procedure—Surgical history	5.0	7.2 (3.1, 15.5)	3.05	21.96 (9.46, 47.28)
Total resources	1248.8	1515.7 (445.3, 3222.5)	14.12	21395.80 (6287.64, 45501.70)

CDA, Clinical document architecture; LDA, Lines drains and airways.

### 2.4 Data query response time analysis

Given that the FDA’s current process to request and receive additional data for a potential adverse event case post-vaccination can take multiple days to weeks, the time to query for EHR records for a potential case is an outcome of interest. We measured the time to query and receive data for each individual patient case through the exchange onto the BEST pilot platform using event timestamps. The measured time included the time taken for the platform to send the query to eHX, eHX to query data from any partners’ FHIR APIs in the state queried, the response time from these partner(s) FHIR endpoints, a delay managed by eHX to accumulate partner(s) endpoint responses, the transmission of data from FHIR endpoints to eHX, eHX processing of transmitted data to bundle data, and the transmission of FHIR bundles to load to the BEST pilot platform. Given the number of steps in this process, it is not meant to be a precise estimate or to identify the exact source of any difference in query time for each case, but instead to provide a general idea whether the platform will be an improvement over the current process.

The current process for retrieving additional data for a reported or suspected AE case involves the FDA contacting a health provider with the request, followed by a manual process to connect and submit the data (6), which can take several days to weeks to complete. For the purposes of this study, any queries taking minutes or even an hour would be considered a substantial improvement (24). Results were calculated separately, based on whether the partner was able to provide AE cases with “limited case windows” or if the case was pulled using the “entire study period,” as the probable size discrepancy between the two groups could lead to differences in case data retrieval times. We calculated the average, median, minimum, and maximum times to query the patient AE cases by these two case types.

### 2.5 Data quality assessment

To assess whether the potential AE case data delivered to the BEST pilot platform had the elements requested by a VAERS form for post-vaccination AE surveillance purposes, we performed a data quality analysis. This analysis was based on the framework for evaluating clinical data quality suggested by Kahn et al. (24), which is well established, used throughout the industry, and recommended by clinical research groups such as Observational Health Data Sciences and Informatics (OHDSI) (25) and Patient-Centered Outcomes Research Institute (PCORI) (26). Table 6 gives an outline of the framework.

**Table 6:** Data quality framework (24).

Conformance	Completeness	Plausibility
Do data values adhere to specified standards and formats? Sub-types include Value, Relational, and Computational.	Are variables present and do they contain all recorded values?	Are data values believable? Sub-types include Uniqueness, Atemporal, and Temporal.
*Relevant example: Are resources coded using interoperable code systems?*	*Relevant example: Are vaccine brand or lot numbers captured for all immunization administrations?*	*Relevant example: Does the AE date occur after the patient birth date?*

The Kahn et al. framework does not prescribe individual data tests but describes the types of questions that fit under each topic of conformance, completeness, and plausibility. To develop our unique data quality tests, we created an initial pool based on those implemented by the open source OHDSI Data Quality Dashboard (DQD) tool (27) or automated data quality assessment. The DQD tool has a suite of tests based on the Kahn et al. framework used to assess a data set stored in an Observational Medical Outcomes Partnership (OMOP) Common Data Model (CDM). We translated these DQD tests, when applicable, to assess the same data quality question in FHIR format. We then defined additional data quality tests with clinical subject matter experts (SMEs), based on the data elements that were either required or optional for filling out a post-vaccination safety report for VAERS or for data elements identified by SMEs as being helpful for the review or analysis of an AE case.

Fast Healthcare Interoperability Resources search requests were developed to query the BEST pilot platform’s FHIR server to obtain results for all data quality tests. FHIR search is the primary mechanism used to find and list resource instances in the FHIR specification (28). The reported test results represent the percentage of resources fulfilling the test criteria out of all applicable resources. These tests describe the existing complexity within RWD exchanged in FHIR format for a public health use case. The FHIR search queries for each test can be shared upon request. Test results <100% do not indicate a failure to meet a standard, since we did not define an *a priori* level of completeness to pass the test, given the many valid reasons or specific allowances for a missing data element, expected format, etc., in FHIR data. Instead, for the data quality tests with <100% of resources that did not meet a test’s criteria, we performed a root cause analysis to assess whether this was related to not being required by USCDI/U.S. Core, medical or IT causes at the partner site, or other reasons, valid or invalid.

Each test was assigned a data quality framework category (conformance, completeness, and plausibility) and a priority, as defined by whether the fields being evaluated were required, optional, or helpful for completing a VAERS report. Tests were labeled as “VAERS Required” if they evaluated fields identified on the VAERS form as essential (e.g., Box 2 Date of birth), while data that could be used to fill out all other boxes on the VAERS form was considered “VAERS Optional” (e.g., Box 25 patient ethnicity). “Helpful” data elements could be those not explicitly requested on a VAERS form but identified by our clinical SMEs as useful for analysis or validation of AEs. For example, immunization dose, quantity, or the onset date of an allergy could be helpful for AE case analysis but are not requested by the VAERS form. “VAERS Required” does not mean those data elements are also required by USCDI/U.S. Core. Thus, we expected many, if not all, of the data elements or conformance specifications that are not included in the current USCDI/U.S. Core requirement to fail, which does not reflect upon the USCDI/U.S. Core compliance of EHR vendors or participating data partners.

Lastly, for each data quality test, we also categorized whether the data element or conformance rule was required by the current USCDI exchange (29), the most recent balloted version of the U.S. Core data set (30), and/or Epic’s own FHIR API functionality documentation (31). The most recent balloted versions of USCDI and U.S. Core IGs, v3 and v6.1.1, respectively, represent a desired future set for the FHIR API requirements. This analysis helps to highlight the number of additional data elements needed for the use case that are not currently required by the USCDI and/or future additions of the U.S. Core data set that are not supported by Epic’s FHIR APIs. Note that USCDI data element support is not always binary. Some USCDI data elements have somewhat generic or ambiguous definitions (e.g., Procedures, Assessments and Plan of Treatment, and Health Concerns), and thus support for those elements is a matter of analysis to determine whether some information “counts” as being within the element or not. Given this ambiguity, we avoided making judgment calls on what meets these requirements and assumed data partners were meeting the requirements in these ambiguous situations.

### 2.6 Statistical analyses

We used the descriptive statistics range (maximum and minimum), median, and average to assess the volume and query response time of the data stratified by the study period “limited case window” or the “entire study period.” We did not conduct any inferential tests to compare the two types of cases because of the small sample size of 11 partners. For the data quality analysis, we calculated the percent of applicable resources that met the test for each partner and then displayed the distribution of each partner’s average outcome and the average of all values for each test on a strip plot (32).

## 3 Results

We received, from our pilot partners, demographic information for 843 post-vaccination AE cases, as several partners were able to supply more than 30 cases. Through the BEST pilot platform, we received 283 out of 358 (79.1%) post-vaccination AE cases queried. In a small percentage of cases, we could not receive the data due to “multiple patient matches” or “no patient matches” errors (18.7% combined) and “Break-the-Glass” security permissions errors (2.2%) as described in the methods section. For almost all partners, we were very close to the 30-patient target, with the exception of one partner with 19 “multiple patient matches” resulting in only 11 cases for analysis. Seven of our data partners were able to provide us with a list of all adverse event reactions to the COVID-19 vaccination and the rest of the partners were able to send a list of majority COVID-19 vaccine reactions such that 88% of our cases had the requested COVID-19 adverse event. The other 12% had a mix of Pneumococcal Conjugate Vaccine (PCV) 13; Tetanus, Diphtheria, and Pertussis (TDaP); Measles, Mumps, Rubella, and Varicella (MMRV); Meningococcal Conjugate Vaccine 4 Oligosaccharid (MCV4O); Influenza; and unspecified by data partner. Table 7 displays the cohort demographics for the 283 successfully pulled patients and the case vaccination distribution.

**Table 7:** Demographics of study cohort.

Category	Demographic group	Total patients	
		*N*	%
Total	Total	283	100.0%
Age	Under 5 years	16	5.7%
	5–17 years	16	5.7%
	18–24 years	16	5.7%
	25–44 years	72	25.4%
	45–64 years	95	33.6%
	65 years and above	68	24.0%
	Missing	0	0.0%
Gender	Male	72	25.4%
	Female	210	74.2%
	Unknown	1	0.4%
Race	White	167	59.0%
	Black or African American	37	13.1%
	Asian/Pacific Islander	10	3.5%
	American Indian or Alaska Native	3	1.1%
	Other	38	13.4%
	Unknown	28	9.9%
	Declined to answer	0	0.0%
Ethnicity	Hispanic	29	10.2%
	Non-Hispanic	223	78.8%
	Unknown	31	11.0%
Immunization type	COVID-19	249	88.0%
	Pneumococcal conjugate PCV 13	9	3.2%
	Tdap	8	2.8%
	MMRV	4	1.4%
	Unspecified	3	2.1%
	Meningococcal MCV4O	1	0.4%
	Influenza	6	2.1%

### 3.1 Data volume characterization

The average count of resources per patient case for the analyzed resource types was calculated for each partner. Table 4 indicates the minimum, maximum, median, and average calculated for the partner average resources per case for all “limited case windows” partners and Table 5 contains the same measures for “entire study period” partners. As expected, the average resource per case for “entire study period” partners were larger, given that generally more clinical information is captured over a longer time period. The difference in average resources per case between the two types of partners was driven by differences in average number of Observation labs and Document Reference clinical notes resources per case.

Overall, the median partner average of resources per patient case was a large volume of data 1062.3 resources per case (393.9 minimum, 1510.6 maximum) for “limited case window” cases and 1248.8 resources per case (445.3 minimum, 3222.5 maximum) for “entire study period” cases. As expected, the “entire study period” partners had large variation in average resources per case, driven mostly by a large variation in the quantity of clinical notes, medication requests, lab tests, and vital signs. Across all partners, lab tests demonstrated the highest volume of data with the median partner averaging 89.2 (for “limited case windows” partners) resources per case or 279.0 (for “entire study period” partners) lab test observations per case. We attempted to generate the approximate size in bytes of an average case sent by the minimum, maximum, and average partner. We calculated average size of a resource for each resource type using a sample of 100 resources of that type. Based on this estimate, given that FHIR resources vary in size, the average partner’s average case size was estimated to be 12.4 MB (5.0 MB minimum, 19.0 MB maximum) for “limited case window” partner and 21.4 MB (6.3 MB minimum, 45.5 MB maximum) for “entire study period” cases.

### 3.2 Data query response time analysis

We measured the time to query and receive data through the exchange onto our platform (Table 8). The median time to query a case across the “entire study period” was 9 min 44 s with an interquartile range (IQR) of 12 min 50 s, much longer than the median case query time for the “limited case window” cases (3 min 30 s, 5 min 52 s IQR). However, the longest and shortest queries for both groups were similar, with the maximum query time of slightly under an hour and a half and the minimum query time for both groups being under 1 min.

**Table 8:** Minimum, maximum, range, and median time to query adverse event cases by partner (hours:minutes:seconds).

Case type	Minimum	Maximum	Range	Median
Limited case window	0:00:12	0:53:00	0:52:48	0:03:30
Entire study period	0:00:56	1:28:55	1:27:59	0:09:44

### 3.3 Data quality assessment

**Figure 2:**
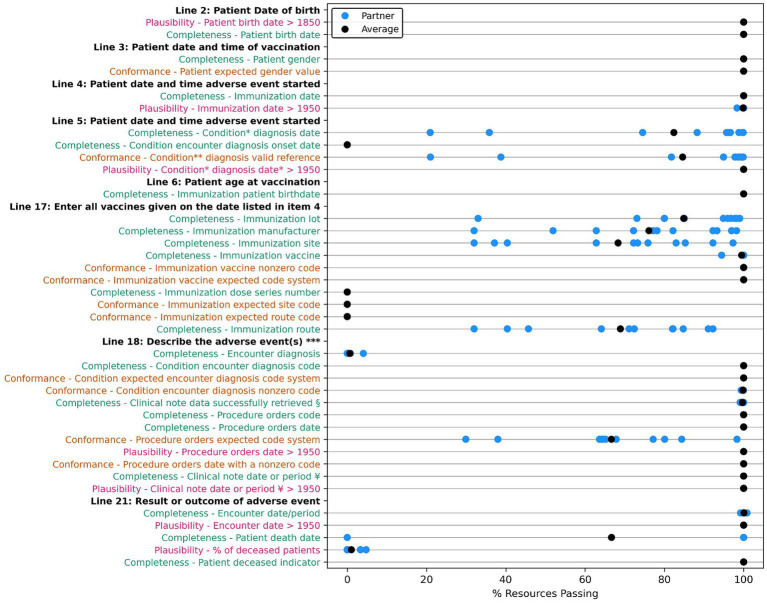
Comparison of data quality results across partners for “VAERS Required” data elements in the VAERS section “Information About the Patient Who Received the Vaccine” (lines 2–6), and section “WHICH VACCINES WERE GIVEN? WHAT HAPPENED TO THE PATIENT?” (lines 17, 18, and 21). Condition * Diagnosis: Condition encounter diagnosis recorded, onset and encounter date. Condition ** diagnosis valid reference: Condition encounter diagnosis valid encounter reference. *** Line 18: Describe the adverse event(s), treatment, and outcomes, if any. § Clinical note data: DocumentReference clinical note data successfully retrieved. ¥ Clinical note date or period: DocumentReference clinical note date or period. Completeness tests are colored green, Conformance tests are colored dark orange, and Plausibility tests are colored pink. Comparison measured by partner average % of resources passing the listed test.

**Figure 3:**
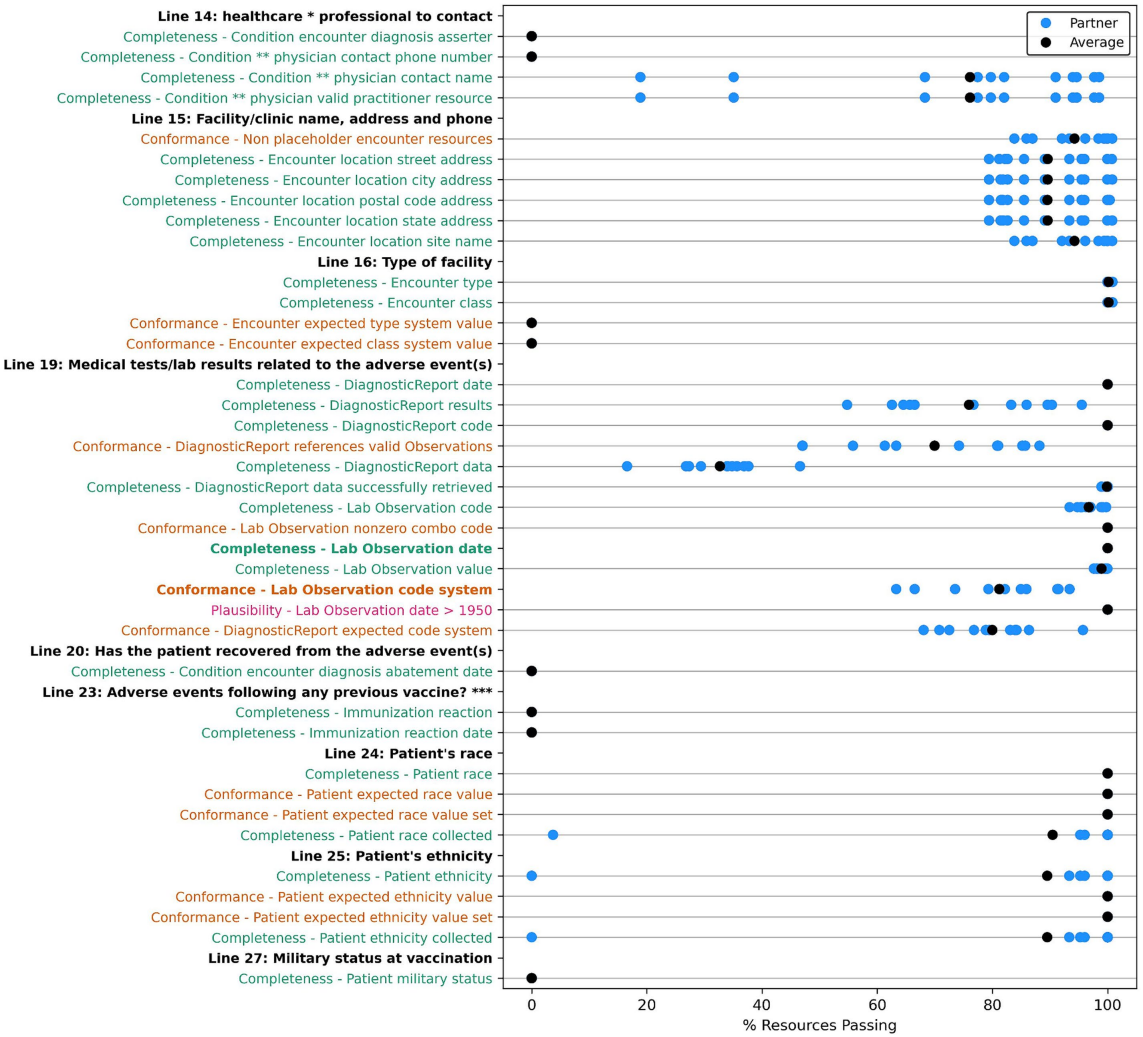
Comparison of data quality results across partners for “VAERS Optional” data elements in the VAERS section: “Information About the Patient Who Received the Vaccine,” Lines 1, 8, 9, 10, and 12. * Line 9: Prescriptions, OTC medications, dietary supplements, or herbal remedies being taken at time of vaccination. ** Line 11: Other illnesses at time of vaccination and up to 1 month prior. Condition ***: Conditions problems/health concerns code. Completeness tests are colored green, Conformance tests are colored dark orange, and Plausibility tests are colored pink. Comparison measured by partner average % of resources passing the listed test.

**Figure 4:**
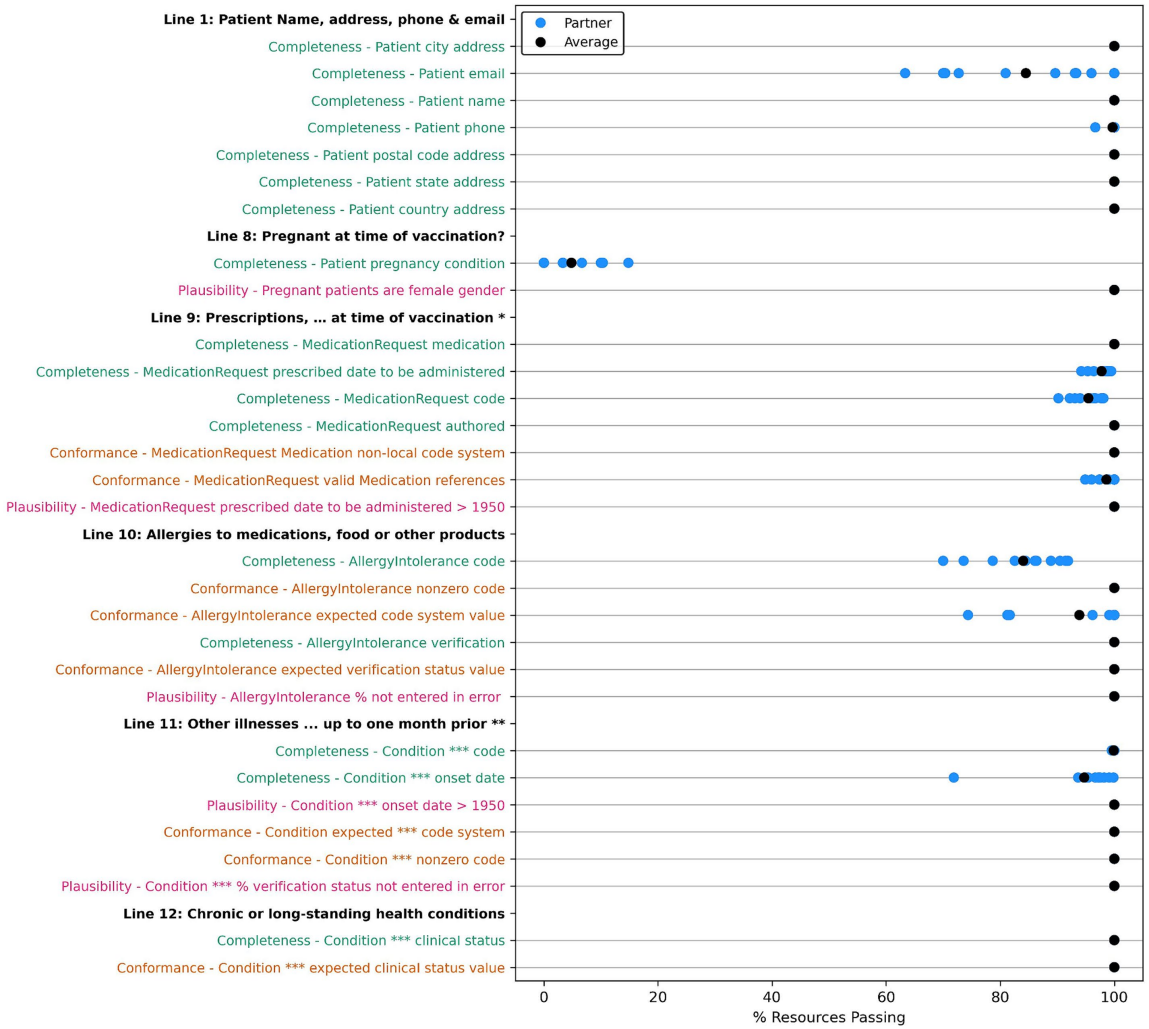
Comparison of data quality results across partners for “VAERS Optional” data elements in the VAERS sections: “Information About the Person Completing the Form,” “Information About the Facility Where Vaccine Was Given,” “Which Vaccine Were Given? What Happened to the Patient?,” “Additional Information,” and “Complete Only for U.S. Military/Department of Defense (DoD) Related Reports.” * Line 14: Best doctor/healthcare professional to contact about the adverse event. Condition ** physician: Condition encounter diagnosis physician. *** Line 23: Has the patient ever had an adverse event following any previous vaccine? Completeness tests are colored green, Conformance tests are colored dark orange, and Plausibility tests are colored pink. Comparison measured by partner average % of resources passing the listed test.

A count of data quality tests for each framework category and priority is outlined in Table 9. For each data quality test, we assessed all applicable resources for all data partners for our group of 283 patient cases. Results are displayed in Figures 2–4 and demonstrate the average percent of resources that passed the test for each partner. Tests are grouped into the VAERS line number to which individual data tests would apply and are further organized by priority and line number. Most of the required fields have 100% of resources passing our data tests. Notable exceptions include condition onset, several of the immunization data elements, patient death date (only measured for deceased patients), and many of the conformance tests.

**Table 9:** Count of data quality tests by priority and category.

	Completeness	Conformance	Plausibility	Total
VAERS “Required”	21	8	10	**39**
VAERS “Optional”	44	7	21	**72**
“Helpful”	112	41	66	**219**
Total	**177**	**56**	**97**	**330**

VAERS, Vaccine adverse event reporting system.

Many of the optional fields have 100% of resources passing our data tests, with exceptions around “contact” and “site type” information for the treating physician, location, and patient in the encounter; allergy, race, and ethnicity; lab test and diagnostic report codes; and many of the conformance tests. For the conformance tests, majority of resources used the prescribed code systems. However, we uncovered some resources without the desired code system, with a missing code or an invalid code value of 0 for AllergyIntolerance, Condition, Medication, and Observation resources. Table 10 shows a summary of the issues unearthed by our root cause analysis for tests with <100% of resources passing with more thorough descriptions of each root cause included in the discussion section. We also compiled the results for each partner for data quality tests for data elements we have categorized as “helpful,” which do not directly populate a field on the VAERS form but are useful for clinical validation or analysis of an adverse event. These results are included in Appendix Figures X1–X13 in the multimedia appendix.

**Table 10:** Root cause analysis findings for data quality tests with less than 100% of resources passing.

Summary of issue
Some necessary data elements are not supported by current or future USCDI/U.S. Core IG requirements.
Immunization data not collected when immunizations are patient-reported or reconciled from outside healthcare provider sites.
Data element uses locally defined, non-interoperable code systems.
Missing information confirming medication compliance.
Technical issues preventing full patient data being pulled into the platform.
Some data elements are not expected to be collected for all resources.
System configuration challenges around patient querying, authorization, and defining API capabilities.

The result of mapping our data tests to existing standards demonstrated that many of the data quality tests are impacted by the data elements not being included in the currently required (v3.1.1) or future (6.1.1) U.S. Core data set as “Must Support” or “Mandatory” and thus often not fully supported by Epic’s FHIR APIs. We analyzed the results to see if the Epic version installed at the data partner site had any effect on the data quality results across partners, but there did not seem to be any obvious correlation between the Epic version and results for individual tests.

Table 11 shows a list of the elements necessary to fill out a VAERS form, and shows if these elements are required by USCDI/U.S. Core IG and the level of Epic’s FHIR API support. Appendix Table 2 within the multimedia appendix shows the list of additional data elements identified as helpful for clinical review but not captured by the U.S. core data set. The mapping shows that several “VAERS Required” or “VAERS Optional” data elements are not required by USCDI/U.S. Core current or future requirements and/or are not supported by Epic’s FHIR APIs.

**Table 11:** USCDI/US Core IG core future requirements and Epic FHIR API support for “VAERS Required or VAERS Optional” data elements not currently required by US Core.

Resource	Data test name	Definition	Priority—VAERS line #	Supported by future US core IG	Epic’s FHIR API Support?
Condition (Encounter diagnosis)	Onset/Recorded/Asserted date	Estimated or actual date of condition onset or first recorded or asserted	Required/Line 5	Y	Not supported
Immunization	Protocol dose number	Dose number within series	Required/Line 17(& 22)	N/A	Not supported
Immunization	Lot number	Vaccine lot number	Required/Line 17(& 22)	N/A	Optional
Immunization	Manufacturer	Vaccine manufacturer	Required/Line 17(& 22)	N/A	Optional
Immunization	Site	Body site vaccine was administered	Required/Line 17(& 22)	N/A	Optional
Immunization	Site value set	Standard site value set	Required/Line 17(& 22)	N/A	Not supported
Immunization	Route	How vaccine entered body	Required/Line 17(& 22)	N/A	Optional
Immunization	Route value set	Standard route value set	Required/Line 17(& 22)	N/A	Not supported
Encounter	Diagnosis	List of diagnoses relevant to the encounter	Required/Line 18	N/A	Optional
Procedure	Performed DateTime	When the procedure was performed	Required/Line 18	Y	Required
Patient	Deceased Boolean	Deceased indicator	Required/Line 21	Y	Required
Patient	Deceased DateTime	Deceased DateTime	Required/Line 21	Y	Optional
Patient	Country	Address country	Optional/Line 1	N/A	Required
Condition	Asserter	Person who asserts this condition	Optional/Line 14	N/A	Not supported
Condition (Encounter Diagnosis)	Abatement date	When in resolution/remission	Optional/Line 20	Y	Not supported
Immunization	Reaction date	When reaction started	Optional/Line 23	N/A	Not supported
Immunization	Reaction	Details of a reaction that follows immunization	Optional/Line 23	N/A	Not supported
Patient	Veterans’ status	US veteran status	Optional/Line 27	N/A	Not supported

USCDI/US Core IG, United States Core Data for Interoperability/ United States U.S. Core Implementation Guide; FHIR, Fast healthcare interoperability resources; API, Application programming interface; VAERS, Vaccine adverse event reporting system.

Of the 31 “VAERS Required” data completeness/conformance tests, 23 (74.2%) have data elements or standards covered by the current and future USCDI/U.S. Core requirements and 27 (87.1%) have some level of support in Epic’s FHIR APIs. Of the 93 “VAERS Required” and “VAERS Optional” completeness/conformance data tests, 79 (84.9.4%) are covered by the current and future USCDI/U.S. Core requirements and 83 (89.2%) are supported to some extent by Epic’s FHIR APIs. Of the total 272 data elements tested for completeness, 216 (78.8%) were covered by the current or future USCDI/U.S. Core requirements, and 241 (88.0%) were supported to some extent by Epic’s FHIR APIs. More promisingly, the mapping also shows that Epic’s FHIR API not only meets all the current requirements but exceeds even the most recent balloted versions of the USCDI (v3)/U.S. Core IG (v6.1.1) by supporting “VAERS Required” and other data elements not required by these versions. Also, Epic’s FHIR API is close to already supporting these future balloted versions for all elements considered in this study, with the only exception being condition encounter diagnosis “onset,” “recorded,” “asserted,” and “abatement” dates.

## 4 Discussion

This study has several interesting and useful findings relevant for any organization that intends to receive and use patient healthcare data from Epic’s FHIR API. We discuss all the results from our analysis in more detail in the sections below:

### 4.1 Data volume characterization

The results demonstrate that, for all partners, the patients had a substantial amount of data even for the “limited case window” cases. Our results show sizable variability in the average volume of FHIR data per case for each partner, especially when pulling patient data for the “entire study period.” The difference in per case data volume between partners could be due to systemic difference between the partners’ data collection methods, clinical processes, or differences in the patient populations supplied to the FDA. Clinical data volume generally correlates to items such as length of stay, number of encounters, or condition complexity. For example, older patients have more data than younger patients, patients with more complex medical issues have more data than the ones with less complex medical issues, and patients with longer inpatient stays have more data than those receiving care in an outpatient care setting. The largest differences between partners are average per-patient count of clinical notes and labs as FHIR resources.

The data volume analysis can help organizations when designing solutions to manage the number of different clinical events, the variability of patient case sizes, and the large amount of data received. The larger the patient case, the more system processing burden on partners, the exchange, and our platform’s infrastructure and applications. Large patient cases also contribute to the challenge of conducting an effective clinical review, as important evidence may be more difficult to find. Therefore, techniques to reduce data received for large cases would be helpful to improve processing time and reduce system burden as long as no pertinent data for clinical reviews is filtered. Further investigation about best practices to reduce the volume of data while maintaining the critical data necessary for clinical review is warranted.

### 4.2 Data query response time analysis

The data query response time analysis demonstrated that the BEST pilot platform represents an improvement over the FDA’s current processes for acquiring follow-up information for a potential AE case review. While there are no estimates for the time it takes to request and receive follow-up information for a VAERS report, the current process requires manual work from the FDA and healthcare providers, has limitations on the data that can be sent, and may require the healthcare provider to complete additional data translation to create a usable format for the FDA (6). As expected, due to the smaller average case size, the “limited case window” generally queried patients faster, but both “limited case window” and “entire study period” cases had outlier values, which took around an hour to query and receive data. As the queries showed improvement over the current process, further analysis on partner differences for query response time was not critical to the goals of this study. Possible causes for variability between patient cases and partner averages include:

1. Larger patient cases take longer to query and load data.
2. Partners use different releases of the Epic software. The earlier releases may have known issues slowing down the API requests that were fixed in subsequent releases/patches.
3. Data partner sites have different backend infrastructure, including different types (e.g., cloud vs. on-premise) of hardware resources, availability, or internet bandwidth.
4. The patient populations vary at data partner’s sites, which can lead to larger databases and increased query time.
5. Potential bottleneck issues with other aspects of the request for information process outside of querying the partners’ FHIR API (e.g., passing the query from the BEST platform to eHX, loading the data back into the platform, querying multiple partners if they are in the same state).

Future research could attempt to isolate the individual components to understand where there are bottlenecks in the process and the cause of differences in partners’ query response time to provide a more complete picture of the speed with which the platform or a similar system can query and receive patient cases. Lastly, future research could test the use of bulk FHIR functionality, an emerging technology that allows a client to request bulk clinical data for a “group” consisting of a set of patients (33) that could allow organizations to receive data more efficiently.

### 4.3 Data quality assessment

Finding that our Epic FHIR API sites exceed the current and future USCDI/U.S. Core requirements is encouraging for the use of the data for postmarket surveillance. However, the results of our data quality assessment identified several tests with less than 100% of resources passing. The root cause analysis results identified that many of the failing tests had similar root causes, which are listed in Table 10 and are described in more detail below.

#### 4.3.1 Some necessary data elements are not supported by current or future USCDI/U.S. core IG requirements

Listed in Appendix Table 2 in the attached multimedia appendix, many of the data elements for filling a VAERS form, especially immunization data elements (e.g., vaccine lot, manufacturer, body site, route, dose series, and reaction) that are not listed as “Mandatory” or “Must Support” by U.S. Core and, for non-VAERS data elements considered “helpful,” were not found in our data. Given there is no obligation to include these data elements in their FHIR resources, Epic’s FHIR API does not support many of them, consistent with the current regulations. USCDI does expand annually to keep pace with clinical, technology, and policy changes creating new draft versions of the USCDI that support additional data elements (34), which could eventually be required for certification via additional regulation. The process to finalize these draft versions, change the certification regulation, and have providers implement could take several years. Therefore, we wanted to review the Epic support for the data elements desired for adverse event validation and reporting that are not listed as at least “Must Support” by U.S. Core IG v3.1.1 for the currently required version of USCDI (35) and identify if they are required by US Core IG v6.1.1 for a the v3 of the USCDI (36). These findings are included in the “Epic’s FHIR API Support” column in Table 2.

Many of these elements such as immunization lot number, manufacturer, site, route, and patient address country are still supported to some extent by Epic’s FHIR API. This presence of immunization data is a key positive result for this study since immunization data are required for vaccine safety surveillance, but immunization lot number has only just been included in the latest draft version of the USCDI (v5) and is years away from being a required field (34). However, a majority of the data elements missing from the USCDI/U.S. Core current requirements are not supported by Epic’s FHIR API based on our results and the Epic documentation (31), including immunization dose number, reaction, U.S. Veterans’ status, etc. Immunization dose series is used to populate VAERS Line 17 (“Enter all vaccines given on the date…Dose Number in Series”) and is required for submission of the form. These elements were not found in any of the resources that we tested across partners.

In addition to supporting data elements that are absent from the current USCDI/U.S. Core requirements, we found that Epic has optional or required support for many of the future USCDI (v3) (36) or U.S. Core IG data (v6.1.1) (37) requirements not incorporated in the current version (v3.1.1) including, but not limited to: condition encounter diagnosis encounter, date, and time a procedure is performed, and patient date of death. The only VAERS required or optional elements that are mandated by the future USCDI/U.S. Core standards that Epic does not support yet are condition encounter diagnosis “onset,” “recorded,” “asserted,” and “abatement” dates. These dates are used to populate VAERS line 5 (“Date and time adverse event started”), and line 20 (“Has the patient recovered from the adverse event”) and are required for submission of the form. The exact onset of a condition may be crucial to understanding whether the adverse event was or was not caused by a vaccine exposure. We expect Epic will support these elements when the future version of USCDI/U.S. Core requirements is enacted. The entire list of required, optional, or helpful data elements that are not supported by Epic’s FHIR API, along with whether or not they are required by USCDI/U.S. Core, is listed in Appendix Table 2 in the multimedia appendix. Our mapping of each data test to being USCDI/U.S. Core-required and Epic-supported is available upon request.

There are alternative methods to obtain an approximation of the information needed for all of the data elements that are not supported. Some examples include but are not limited to:

- For immunization dose series, a list of historical immunizations ordered by immunization date.
- Most of the encounter diagnosis conditions reference an encounter that has a start and end date populated.
- Clinical notes captured in Document Reference resources include narratives about the case that can fill in many of the blanks left by various missing data elements.

#### 4.3.2 Immunization data not collected when immunizations are patient-reported or reconciled from outside healthcare provider sites

Given the FDA’s vaccine safety use case, we paid special attention to the quality of the immunization resources received from our data partners. The key immunization information sufficient for evaluation of a VAERS report, such as immunization lot number, manufacturer, site, and route (minus the unsupported dose series number) were populated for most resources, but with missing resources. Our analysis showed that this information, especially lot number and manufacturer, was populated for every vaccine administered within the healthcare provider system and almost all of the missing data were traced to either immunizations reconciled from outside sources or patient-reported vaccines. Immunizations reconciled from other providers through vaccine registries almost always included the vaccine lot number, but were sometimes missing site, route, and manufacturer information. Patient-reported immunizations were often missing lot number, manufacturer, site, and route. These findings demonstrate that healthcare providers are able to pull in records of an immunization that occurred outside of the hospital settings through state vaccine registries even when they are missing some critical data. These results in a large data gap, considering the amount of immunizations that are administered outside of a traditional health provider setting (e.g., pharmacies, grocery stores).

We found a large variation in completeness of these elements between data partners, especially manufacturer, site, and route. Because the data issues are caused primarily by external site vaccinations, it is unclear whether this is a partner-specific issue (e.g., the partner records more patient-reported immunizations than other healthcare providers) or a result of who they are receiving patient vaccine data from (e.g., the partner has more patients receive their immunizations from a source that does not collect or transmit full information). Additionally, this could be attributed to variability in integrations with Immunization Information Systems in different United States regions. This issue may be more visible for immunizations over other resources because Epic chose to auto-reconcile (incorporate into the local record) all incoming COVID-19 vaccinations from other organizations, whereas non-COVID-19 immunizations as well as problem lists, medications, and allergies, require manual requests by a caregiver for reconciliation into the local record. As such, during the study period there were much more external data in patient records for COVID-19 immunizations than for any other type of immunization or other resource.

#### 4.3.3 Data element uses locally defined, non-interoperable code systems

One of the key challenges to healthcare data exchange is achieving semantic interoperability, when data are not only exchanged between two systems but also understood by each system. While the U.S. Core requirements prescribe standard code or value sets for most of the applicable data elements in their profiles, they prioritize requiring standardized code sets for the coded value of a resource. This functionality allows a clinician to understand specifics of diagnosis, medication, lab test, or other events of the resource. Almost all resources had valid coded values using the required standard, interoperable code sets. The results for the percentage of non-standard codes or codes systems are listed as different conformance tests in Figure 2. We found that Immunization, DocumentReference, and Patient and Diagnostic Report resources all used valid codes and U.S. Core required code systems. We included the percentage of non-passing tests, along with examples, in Appendix Table 3 in the multimedia appendix, for at least one conformance test with <100%.

The data have larger conformance problems for other non-code data elements, including some in which the current U.S. Core specification only suggests, but does not require, a standard code or value set to use. This finding is consistent across partners, so we suspect it is a systemic issue across the industry, which has not defined, specified, or coalesced around standard value sets or code lists for these elements. These specifications allow for some or all of the codes to be locally defined values/codes through value sets that are extensible (38), preferred (39), or example (40). Example sets are not expected or even encouraged to draw from the specified values (40). For elements such as encounter care setting, immunization site, and route, because there is no requirement to match a specified code or value set, they are always populated with codes defined locally at our partner sites through the Epic EHRs. Each of these fields has a text description that can be easily understood by a clinician, but the lack of a standardized code system can cause difficulties for any simple machine processing or algorithmic use of the data. Future resources could analyze the ability of NLP techniques to identify similar values across data partners and map them to an expected code system or value set. Additionally, future versions of the USCDI or U.S. Core could change the requirement to defined required code lists, using published code lists for the different elements like Immunization site and route value sets defined by FDA and CDC for VAERS reporting Appendix Table 4 in the multimedia appendix demonstrates details of fields that are populated with only locally defined codes (41).

#### 4.3.4 Missing information confirming medication compliance

Patient medication information is needed to fill out VAERS line 9: “Prescriptions, over-the-counter medications, dietary supplements, or herbal remedies being taken at the time of vaccination” and to assess other potential causes/interactions for the adverse event. Because the Medication domain in the FHIR standard includes a number of related resources, it is difficult to assess whether the necessary data elements are populated. Some medication resources that might apply to our use case include:

- MedicationRequest: Used for an order for both supply of the medication and the instructions for administration of the medicine to a patient (42).
- MedicationDispense: The provision of a supply of a medication with the intention that it is subsequently consumed by a patient (usually in response to a prescription, order, or request).
- MedicationAdministration: When a patient consumes a medicine, or it is otherwise administered to them.
- MedicationStatement: A record of medication being taken by a patient, or that the medication has been given to a patient where the record is the result of a report from the patient, or another clinician.

Currently, the required version (3.1.1) of the U.S. Core IG mandates that MedicationRequest resource be populated but amends the definition of MedicationRequest to be able to return all prescribed and “self-prescribed” medications directly ordered by a provider or reported by the provider, patient, or related person including ethical drugs, over the counter (OTC) medication, and other substances taken for medical and/or recreational use. This ensures “self-prescribed” medications are captured in MedicationRequest as opposed to other resources, such as MedicationStatement. However, this definition does not convey compliance to the prescription (43), so in cases in which the patient does not comply (or does not comply at the prescribed time), it can be difficult to know exactly what medications were being taken at the time of vaccination. Future versions of the U.S. Core include the MedicationDispense profile (37), which could address concerns about including medication compliance information and better support this FDA use case as well as other drug safety and effectiveness use cases.

#### 4.3.5 Technical issues preventing full patient data pulled into the platform

We identified two technical issues that prevented us from pulling in data for tests around referenced resources or large free text data elements. Although the data are available from the partner FHIR APIs for these resources, they were not pulled into our platform because of the reasons listed below.

##### 4.3.5.1 Reference chasing

All FHIR resources can reference other resources. To obtain all necessary clinical information for the FDA’s use case, we had to pull referenced resources that may not be specifically linked to the patient resource (e.g., the location of patient’s encounter, or the contact information from the practitioner that diagnosed the patient’s AE). Because these referenced resources can also allude to other resources, eHX added a limit to the level of reference chasing when pulling information from healthcare FHIR APIs. eHX only pulled resources that reference the patient queried or are referenced by any of the resources that reference the patient queried. However, we suspect this led to missing data for the AE encounter location, as an initial location resource can reference a parent location (e.g., “Data Partner 1 Emergency Medicine Wing” Location could be part of the “Data Partner 1 Hospital System” location) and the address information may only be captured with the parent location due to the reference chasing limit. This is an imposed limitation of the BEST pilot platform and eHX so the test results may not reflect the total data available through a data partner’s Epic FHIR API.

##### 4.3.5.2 API time out errors

When querying some partners’ APIs, we observed timeouts when requesting Document Reference resources with large clinical notes. These timeouts would result in some of the data failing to be received by eHX and thus, our platform. We worked with our partners and their Epic contacts to identify and apply a fix that greatly reduced or eliminated the amount of missing data. The results of the “Completeness–document reference data successfully retrieved” test under the VAERS Line 18 section demonstrates clinical note text for only a small fraction (average % missing across partners: <0.3%) missing.

#### 4.3.6 Some data elements are not expected to be collected for all resources

For data elements labeled “Must Support” by the U.S. Core requirements, we observed less than 100% of resources with the element populated. A “Must Support” U.S. Core data element requires responders to be capable of populating all data elements and processing resource instances but does not require populating the data element if it is not present. In contrast, a “Mandatory” data element is expected to be populated for every individual resource. This indicates that some “Must Support” data elements will not always be populated for some or even all resources, as an element may not apply to the type of clinical data collected in our sample or is missing due to the element’s natural rarity in the population. For example, the death date element is missing for many to all of our patient resources because only a small percent of the population will be deceased, and it may not have appeared in our sample naturally. Further investigation of larger or targeted samples of patients is needed to confirm that these data elements are populated for all partners. Missing data elements also occur in resources when a small but expected percentage of clinical events will be missing data, for example an allergy may be missing a code if it is for an unusual substance not captured in the code system or patients may be missing an email address if they do not report it to the hospital system.

### 4.4 System configuration challenges around patient querying, authorization, and defining API capabilities

Across the different analyses and characterizations, there were several technical, system configuration, or authorization challenges due to variability in partners’ FHIR implementations that delayed our ability to connect to the FHIR APIs and pull the necessary patient cases. Configuration or authorization issues could expand the time it took for a partner to connect to the system from a single day to 1–2 weeks of intermittent troubleshooting sessions. When these issues prevent patient cases from being able to be queried, it removes potentially valuable information for FDA reviewers. Technical issues included those indicated below.

#### 4.4.1 Partner API configuration can prevent some patient queries

Some patients are unable to be queried due to multiple patient matches and “Break-the-Glass” security protections described in Methods: Data section above. Multiple patient matches occur when the demographics provided in the patient search queries match more than one patient in the healthcare organization. The patient search operation was designed for a system with an end user who can evaluate the list of patients returned (44) to select the specific patient of interest. Due to patient privacy concerns, our platform rejects multiple patients to ensure we are not exposed to patients without a probable post-vaccination adverse event. Because FHIR search operation allows for an organization to configure a specified amount of variance in the patient’s demographics and still return a match (45), the amount of multiple patient matches could vary by partner and could be a sizable portion of patients queried if a high amount of variance is allowed.

Epic has released a new FHIR $match operation designed to find a single, high quality patient match (44). This operation was not released and readily available at all partner FHIR endpoints in time for our study but could be applied to eHX patient query in the future. Some data partners have reported better querying performance using Patient.$match (44) instead of Patient.Search (45) to complete the demographic search for cases. For “Break-the-Glass,” an override is possible, and we successfully deployed a web service with one partner that could override these protections by creating a unique “service account” that has access to these patients. However, this solution would need to be distributed and reviewed with the rest of our partners.

#### 4.4.2 Partner system configuration settings and processes for authorizing queries/capabilities

While setting up our initial connection to partner FHIR APIs, we encountered configuration and authorization issues. The eHX team planned to seek authorization from the data partners’ IT contacts and automatically check that we had all the necessary authorization by querying the API for the FHIR capability statement. This statement should be provided for every FHIR API and describes the actual functionality for the user (46). Unfortunately, because of Epic’s two-tiered security model, in which a user needs access approved for both the API and the backend EHR database, the backend security model may deny access to FHIR resource types listed as functional for the user in the capability statement. Therefore, the only way to confirm a connection for each partner’s FHIR endpoint is to work iteratively with the data partners’ IT teams to discover the approvals needed and then manually test and adjust until all data are successfully able to be pulled.

The authorization process varied across our data partners despite using the same EHR software. Our partners often had no internal standard process for this authorization but approvals from one or more partner teams and multiple stakeholders, such as the partner’s health information management team and a dedicated security team, were sought. At some partners, the process can involve seeking authorization at the resource level (e.g., Condition or DocumentReference), while others require separate authorization to query for each of the subcategories (e.g., condition problem statement, condition health concern, etc.) of a given resource. These were minor issues that could be resolved in a few hours or days depending on the partner. However, the effort and time required from both the partner and eHX staff reduced the ability of the solution to quickly add new partners and scale to the necessary population size.

Some improvements could be made in this study to speed up the process of connecting and receiving authorization to Epic’s FHIR APIs at future partner sites. Because these issues are due to the higher-level reason of custom categories and variability in requiring type-level access, a potential solution is for each data partner to have a direct FHIR interface allowing for automated client registration based on the profile of the FHIR client. For example, a FHIR client requesting data access for a “public health” purpose would be granted specific data access based on that purpose. The Office of the National Coordinator for Health Information Technology (ONC) is considering requiring this in the future and could cite this as evidence for this requirement given that these issues may affect other projects using FHIR endpoint connections for health information exchange.

### 4.5 Methodologic considerations

This study is a novel approach and one of the first attempts to characterize the FHIR APIs’ data volume, query response time, quality, and extent to which they meet USCDI/U.S. Core requirements after the ONC final rule requirements were enacted. Other strengths of this study include the size of the sample of real-world patient case data (283 patients) across different data providers (11). Given the variability in individual patients, a smaller sample size would make it impossible to distinguish if variation in results for different partners is due to actual variation between the partners FHIR API capabilities or differences between the patient populations pulled. Furthermore, this study is robust because of the amount of analysis completed on the USCDI/U.S. Core and Epic FHIR documentation for fields that map to the VAERS form. Using the data quality framework, the study also demonstrated the utility of using existing data quality frameworks applied to FHIR standard. It is our hope that this analysis can help guide the development of future USCDI/U.S. Core requirements and be used by others with similar public health use cases.

#### 4.5.1 Study limitations

Some limitations of the study include the non-random samples of healthcare organization data partners and limited focus on the FDA use case. The data partners that volunteered for the study are likely not representative of healthcare organizations across the country. They are among the larger and more technically-sophisticated healthcare organizations, given their previous connection to the eHealth exchange and their current capacity to support the FDA’s BEST pilot platform. Another weakness of our study is that it has limited power to explain the variability observed in the data. Future research could stratify patients based on key data elements such as age, medical complexity, care setting, or other variables, which could offer valuable insights around observed variability. Also, the study only focused on resources and data elements relevant to the FDA use case. Additional analyses would be needed to evaluate how well the exchange solution is able to receive data for other clinical use cases.

Lastly, all of our data partners use the same EHR vendor, Epic EHR, suggesting that our analysis could overstate or understate the data quality, volume, or query response time of the data received when compared to the population of all United States healthcare providers. Participants that used other EHR vendors had multiple barriers to participating, including many factors such as technical feasibility, financial, administrative, or other unspecified reasons to not participate in the study. Additionally, smaller provider organizations may not have as much bandwidth to support the pilot compared to the larger organizations that engaged with us. Future research could complete our USCDI/U.S. Core requirements mapping using another vendor’s documentation or redo the entire data volume, query response time, and quality analyses on other data partners with a different EHR vendor which would more clearly demonstrate the true operational challenges and state of the industry with implementation of FHIR interoperability in EHRs since the biggest challenges will be cross system. Since Epic is the largest EHR provider and holds significant market share (47), we believe our analysis still is a valuable first step.

### 4.6 Conclusion

This study demonstrated that ONC’s new USCDI data requirements have created a feasible technical alternative for public health entities to access a large set of EHR data across many healthcare provider partners. Our experience creating and assessing our healthcare exchange-based pilot platform was able to confirm the feasibility of this approach, but also identified several challenges for developing a live, nationwide active surveillance system for the FDA. The study answered the questions posed in the introduction:

1. Are the data elements received from EHR FHIR endpoints sufficient for FDA’s vaccine postmarket surveillance activities? The EHR data elements found in the FHIR resources had plausible values and mostly conformed with the standards involved with filling out a VAERS report for a vaccine adverse event. All the current USCDI/U.S. Core requirements are supported by Epic’s FHIR APIs, and the APIs also had a degree of support for data elements that are not yet required or that will be required in the future. This is an encouraging sign for interoperable data exchange as it shows that at least one EHR vendor supports FHIR capabilities beyond USCDI/U.S. Core requirements. However, there are several vaccine AE-related data elements, including those for immunization data and condition dates that are not required by the current version of the USCDI and thus are often missing from the received EHR data. The information gathered around these gaps can inform other parties that require this information and spur a conversation around improvements to future versions of the USCDI and U.S. Core requirements for EHR vendors’ API implementation.
2. What is the quantity and type of data available? The data volume analysis demonstrated how substantial volume and size of data can be received for a vaccine AE patient case. Specifically, most of the volume of FHIR resources for a case consists of clinical notes, medications, condition diagnosis, and lab tests/vital sign observations. We found a median average resource count per partner of 983.3 semantically-relevant clinical events and an estimated size of 12 MB within our “limited case window” (i.e., from immunization date through 10 days after the end of the encounter with the adverse event diagnosis—around a month for many cases). This finding will need to be considered when designing a larger-scale production system both for the technical challenges for data volume as well as the need for clinical reviewers to have tools to accelerate chart reviews of large cases. Any implementer of exchange-based solutions for case reporting or analysis should have a plan for storing, filtering, and allowing users to view and search through the case data.
3. How fast can the data be received through a health information exchange using FHIR, such as the BEST pilot platform? Almost all cases are received through the exchange within minutes, but occasionally take over an hour. This is still a substantial improvement over existing manual processes for the FDA.
4. How easy is it to onboard a new partner to the exchange platform? The process of onboarding a partner to send FHIR data through a HIE could be improved by a standardized process for giving permission and security approval for their FHIR API once a data partner has approved the connection to the healthcare data exchange.

Overall, these analyses show that interoperable EHR RWD is of sufficient quality to support an active surveillance system that informs post-authorization regulatory responsibilities of agencies such as the FDA.

## Data availability statement

The data analyzed in this study are subject to the following licenses/restrictions: data were available to the FDA for the purpose of reviewing potential adverse events post-biologic exposure and are not available as a de-identified public data set. Inquires about these datasets should be directed to HE, Hussein.ezzeldin@fda.hhs.gov.

## Ethics statement

Ethical approval was not required for the study involving humans in accordance with the local legislation and institutional requirements. Written informed consent to participate in this study was not required from the participants or the participants’ legal guardians/next of kin in accordance with the national legislation and the institutional requirements.

## Author contributions

MD: Conceptualization, Data curation, Formal analysis, Investigation, Methodology, Software, Supervision, Visualization, Writing – original draft, Writing – review & editing. RD: Resources, Validation, Writing – review & editing, Data curation, Investigation, Methodology. LJ: Conceptualization, Project administration, Supervision, Writing – review & editing. AS: Data curation, Formal analysis, Software, Visualization, Writing – original draft. BG: Data curation, Formal analysis, Visualization, Writing – review & editing. AP: Investigation, Resources, Writing – review & editing. SC: Writing – review & editing. RF: Writing – review & editing, Supervision. SA: Conceptualization, Funding acquisition, Writing – review & editing. HE: Conceptualization, Funding acquisition, Project administration, Supervision, Writing – review & editing.
