# Supplementary material for: Data quality and timeliness analysis for post-vaccination adverse event cases reported through healthcare data exchange to FDA BEST pilot platform
Source: Front Public Health. 2024 Jul 8;12:1379973. doi: 10.3389/fpubh.2024.1379973 (PMC11260708; doi:10.3389/fpubh.2024.1379973)
Supplement: Supplementary file 1 [file Data_Sheet_1.docx]

**Data Quality and Timeliness Analysis for Post Vaccination Adverse Event Cases Reported Through Healthcare Data Exchange to FDA BEST Pilot Platform**

*Multimedia Appendix*

**Document Description**

This is a Multimedia Appendix to a full manuscript published in the Frontiers in Public Health-Digital Public Health

**Table 1: List of status values used to identify non semantically relevant resources**

| ***Resource*** | ***Data Element Filter*** | ***Non-Semantically Relevant Statuses*** |
| --- | --- | --- |
| AllergyIntolerance | clinical-status | entered-in-error |
| Condition | verification-status | entered-in-error |
| DiagnosticReport | Status | entered-in-error |
| DocumentReference | status | entered-in-error, superseded |
| Encounter | Status | entered-in-error |
| Immunization | status | entered-in-error, not-done |
| MedicationRequest | status | entered-in-error, cancelled, draft |
| Observation | status | entered-in-error |
| Procedure | status | entered-in-error, not-done |

**Table 2: Missing data elements from USCDI core data set that were identified as helpful for post vaccination AE analysis**

| ***Resource*** | ***Data Test Name*** | ***Definition*** | ***Priority – VAERS line #*** | ***EPIC API Support?*** |
| --- | --- | --- | --- | --- |
| AllergyIntolerance | Category | Category of identified substance associated with allergies (i.e., food, medication, etc.) | Helpful | Required |
| AllergyIntolerance | Date | Date of allergy was recorded | Helpful | Required |
| AllergyIntolerance | Allergy onset date | Date of allergy onset | Helpful | Optional |
| Condition | Body | Anatomical location, if relevant | Helpful | Not Supported |
| DocumentReference | Relates to | Relationships to other documents | Helpful | Optional |
| Immunization | Target disease | Vaccine preventable disease being targeted | Helpful | Not Supported |
| Immunization | Reason | Why immunization occurred | Helpful | Optional |
| Immunization | Dose quantity | Amount of vaccine administered | Helpful | Optional |
| Immunization | Performer | Who performed the immunization | Helpful | Optional |
| MedicationRequest | Dispenser organization | Medication to be dispensed by this Organization | Helpful | Not Supported |
| MedicationRequest | Performer | Intended performer of administration | Helpful | Not Supported |
| MedicationRequest | Priority | Level of importance of actioning request | Helpful | Not Supported |
| Procedure | Performer | Who performed the procedure | Helpful | Optional |
| Procedure | Reason | Reason procedure performed | Helpful | Optional |

**Table 3: Resources with invalid coded values**

| ***Resource*** | ***% invalid code (avg.)*** | ***Explanation*** | ***Priority – VAERS line #*** |
| --- | --- | --- | --- |
| Condition | <0.2% | Resources have no codes when they are category genomic indicators, and some partners use a SNOMED invalid value of 0 to indicate an erroneous encounter. | Required - Line 18: Describe the adverse event(s),… treatment, and outcomes, if any |
| Medication Request | 6.2% | A small number of medications do not have any coded values. | Optional - Line 9: Prescriptions, OTC medications, etc. being taken at time of vaccination |
| Observation | 3.5% | Small % of observations are missing codes and others only use proprietary codes systems. Further investigation is needed. | Helpful |
| Allergy Intolerance | 16.1% | Missing codes are caused by either allergies to substances that are not listed as coded values or a record of “No known allergies”. | Optional - Line 10: Allergies to medications, food or other products |
| Procedure | 43.6% | Surgical history procedures are missing codes, although USCDI requires code for all procedures. | Optional - Line 18: Describe the adverse event(s), treatment, and outcomes, if any |

**Table 4: Data elements with only local code systems or value sets**

| ***Resource*** | ***Data Element*** | ***Example**** | ***Expected Code or value set*** | ***Priority – VAERS line #*** |
| --- | --- | --- | --- | --- |
| Encounter | Type | "system": "urn:oid:1.2.840.114350.XXXXX",  "code": "102”,  “display”: “Outpatient" | US-Core Type ([1](#_ENREF_1)) | Optional - Line 16: Type of facility |
| Encounter | Class | "system": "urn:oid:1.2.840.114350.XXXXX",  "code": "17",  "display": "Requisition Encounter" | hl7 enc. Class ([2](#_ENREF_2)) | Optional - Line 16: Type of facility |
| Immunization | Site | "system": "urn:oid:1.2.840.114350.XXXXX",  "code": "6”,  “display”: “Right deltoid" | hl7 imm. site ([3](#_ENREF_3)) | Required - Line 17(&22): Enter all vaccines given on the date listed in item 4 |
| Immunization | Route | "system": "urn:oid:1.2.840.114350.XXXXX",  "code": "2”,  “display”: “Intramuscular" | hl7 imm route ([3](#_ENREF_3)) | Required - Line 17(&22): Enter all vaccines given on the date listed in item 4 |

** Synthetic test data used*

**Figure X1: Data Quality Results for Helpful Data Tests Applicable to AllergyIntolerance FHIR Resource**

**
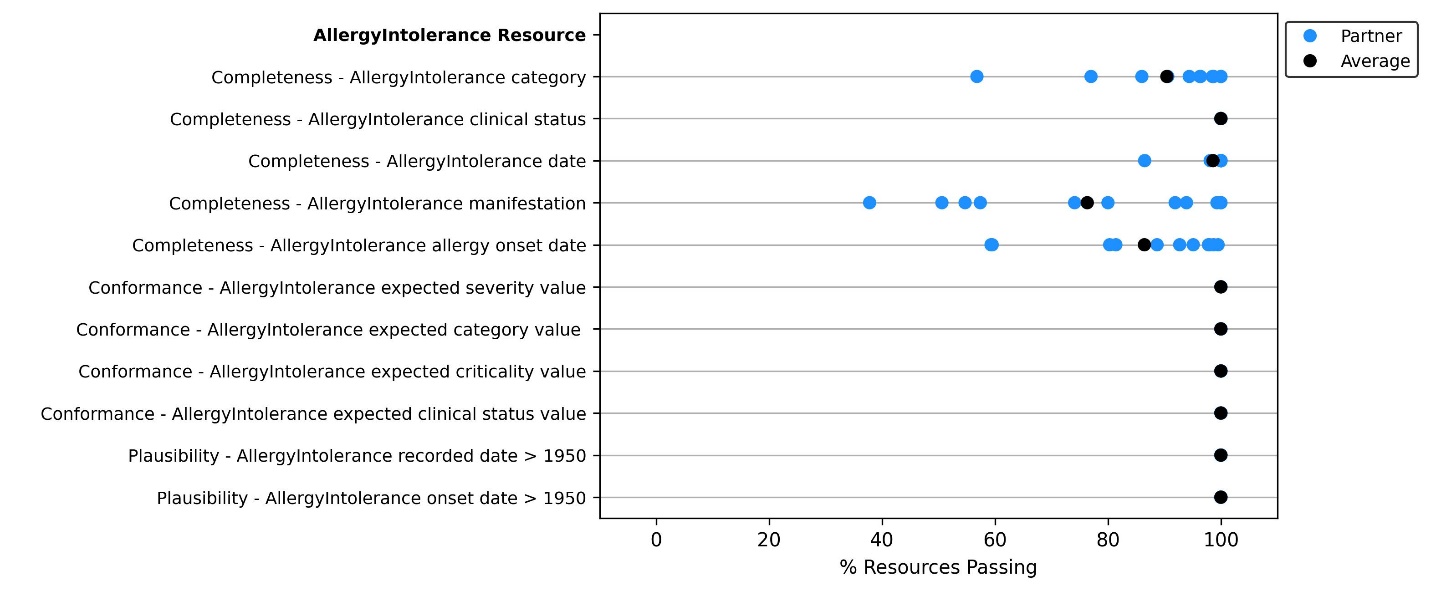
**

Figure X1. Comparison measured by partner average % of resources passing the listed test.

**Figure X2: Data Quality Results for Helpful Data Tests Applicable to Condition FHIR Resource (Completeness)**

**
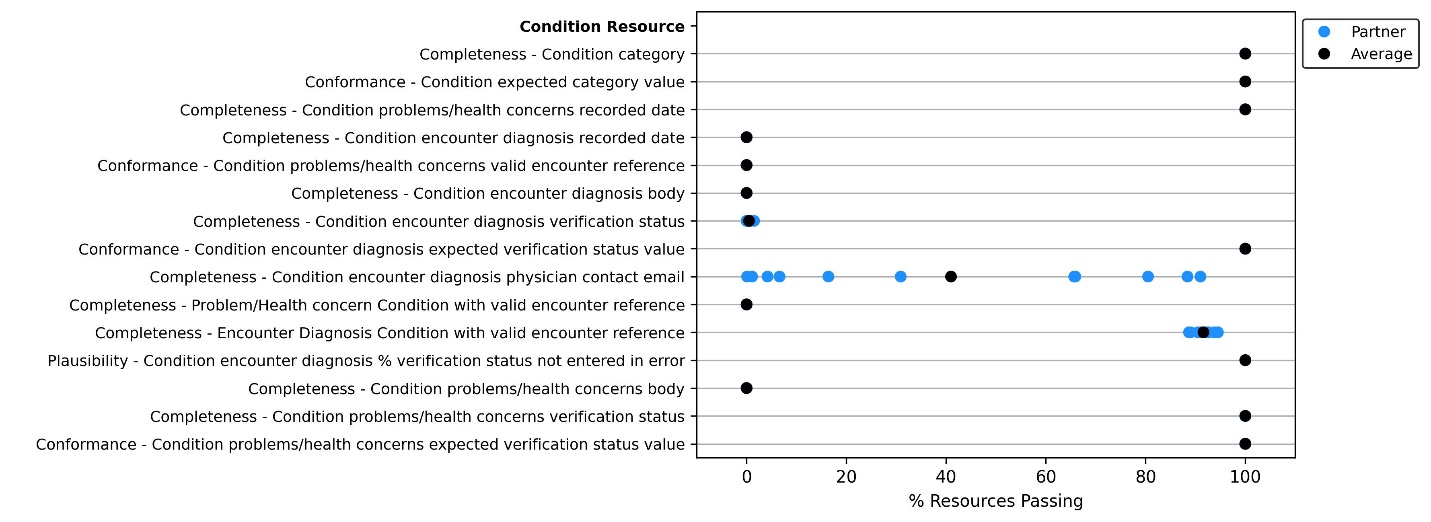
**

Figure X2. Comparison measured by partner average % of resources passing the listed test.

**Figure X3: Data Quality Results for Helpful Data Tests Applicable to DocumentRefernce Resource**

**
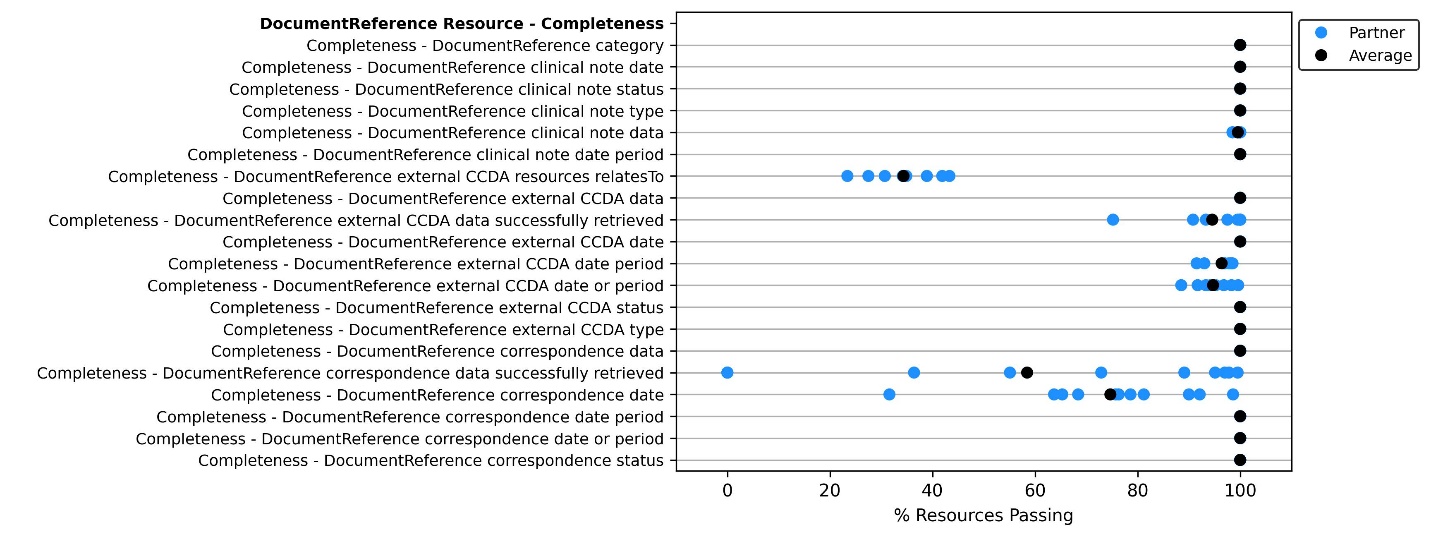
**

Figure X3. Comparison measured by partner average % of resources passing the listed test.

**Figure X4: Data Quality Results for Helpful Data Tests Applicable to DocumentRefernce Resource (Completeness - cont.)**

**
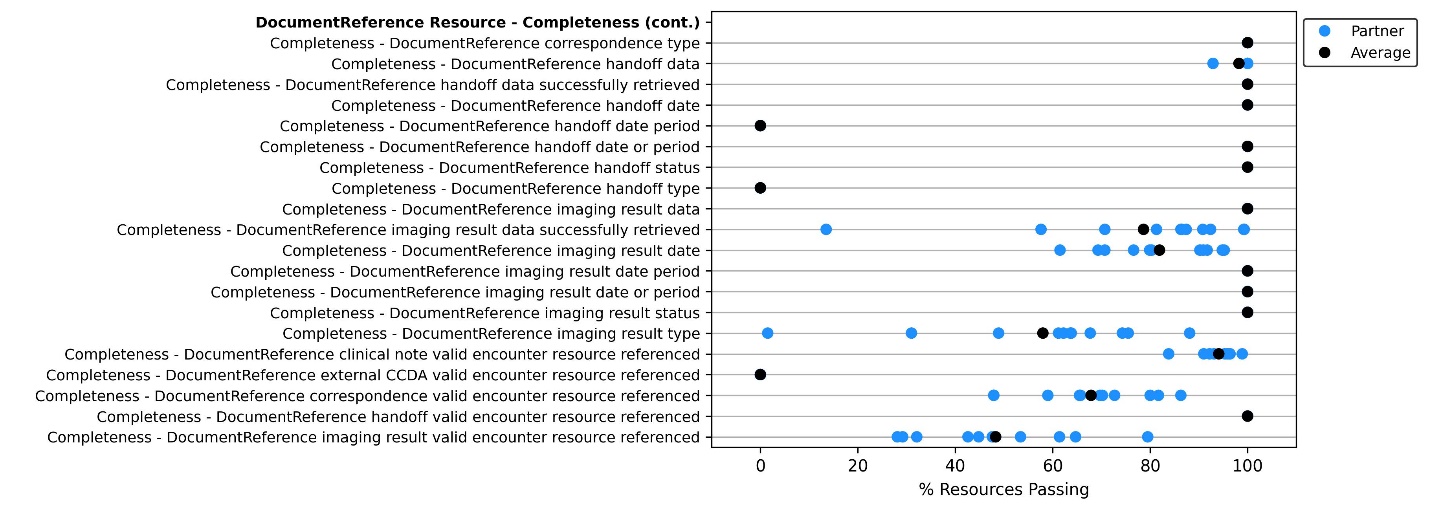
**

Figure X4. Comparison measured by partner average % of resources passing the listed test.

**Figure X5: Data Quality Results for Helpful Data Tests Applicable to Condition FHIR Resource (Completeness)**

**
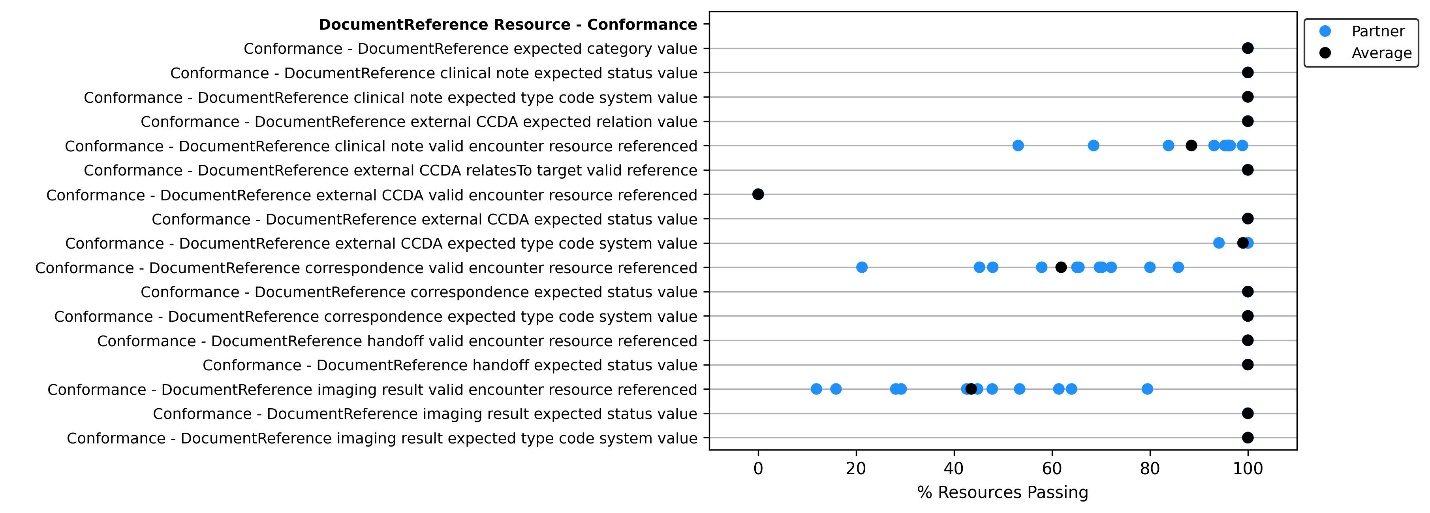
**

Figure X5. Comparison measured by partner average % of resources passing the listed test.

**Figure X6: Data Quality Results for Helpful Data Tests Applicable to DocumentReference Resource (Plausibility)**

**
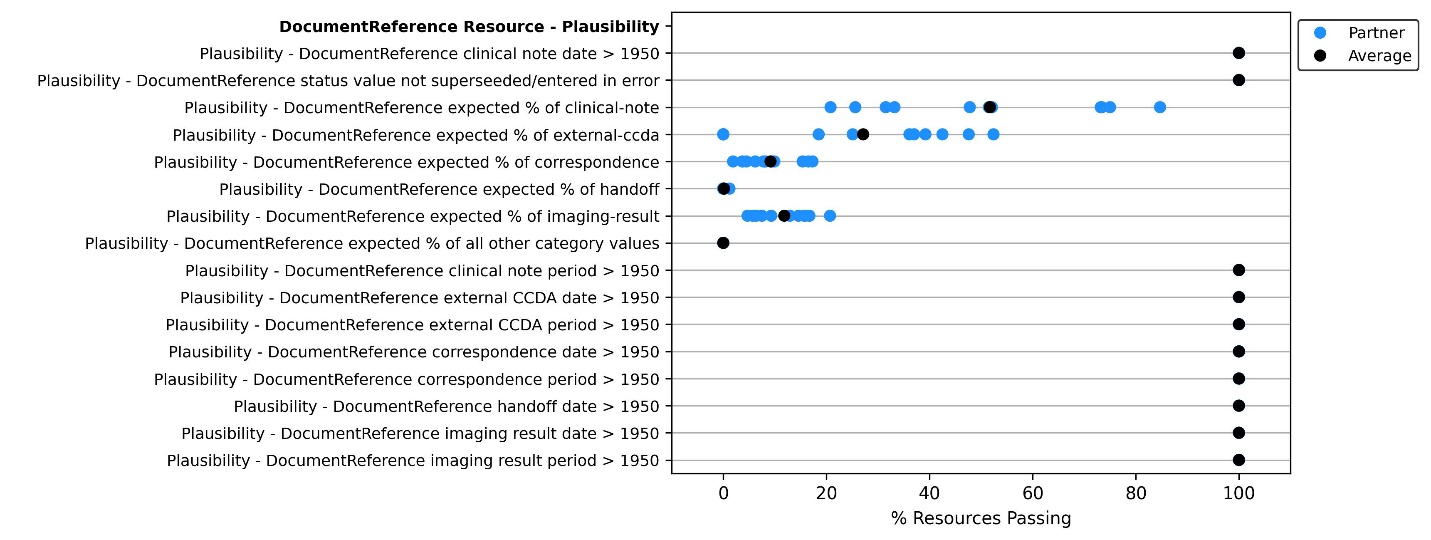
**

Figure X6. Comparison measured by partner average % of resources passing the listed test.

**Figure X7: Data Quality Results for Helpful Data Tests Applicable to Encounter Resource**

**
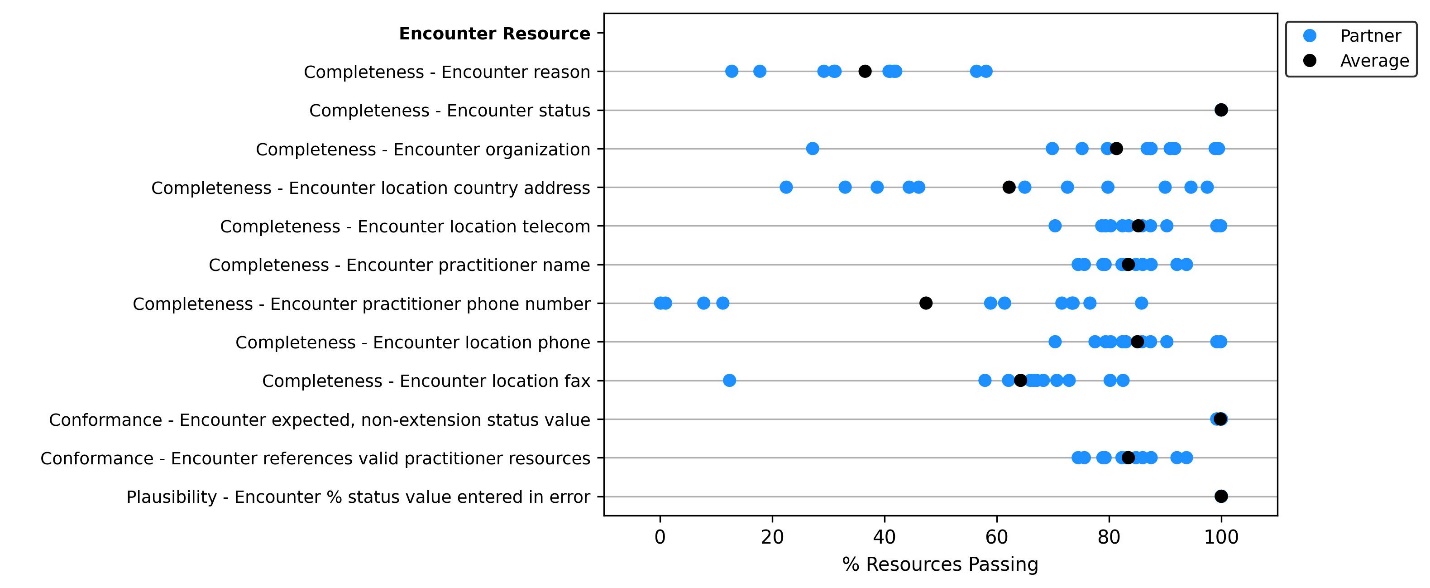
**

Figure X7. Comparison measured by partner average % of resources passing the listed test.

**Figure X8: Data Quality Results for Helpful Data Tests Applicable to Immunization Resource**

**
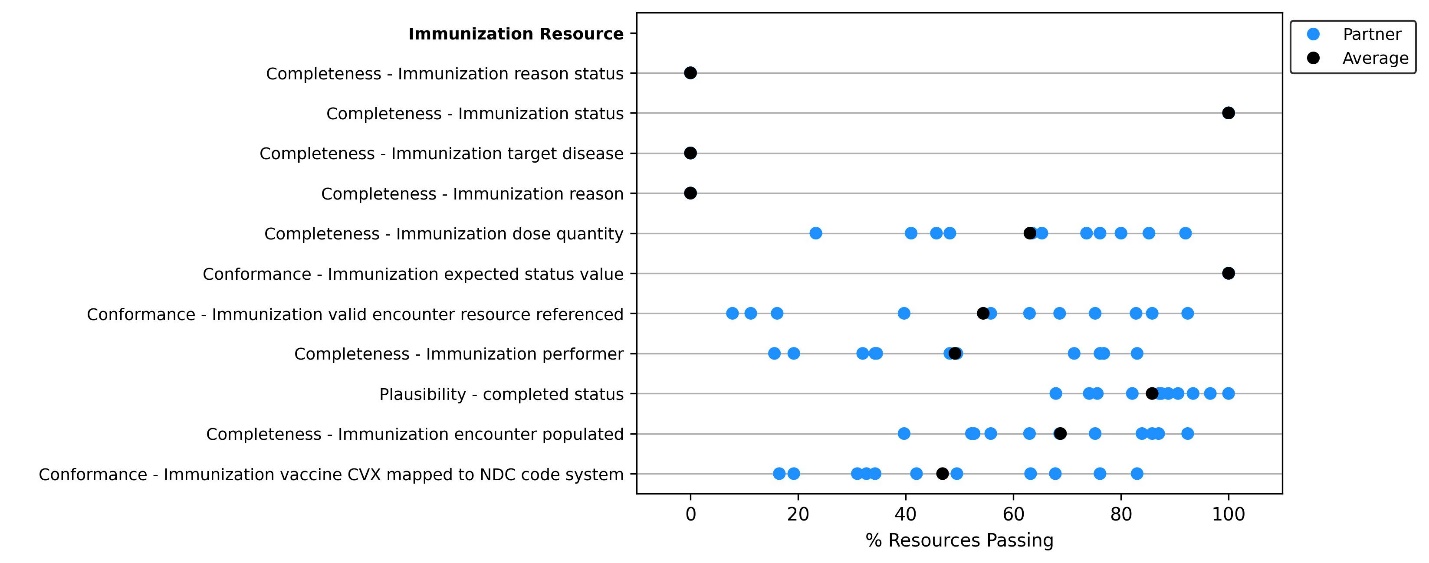
**

Figure X8. Comparison measured by partner average % of resources passing the listed test.

**Figure X9: Data Quality Results for Helpful Data Tests Applicable to Observation Resource (Completeness)**

**
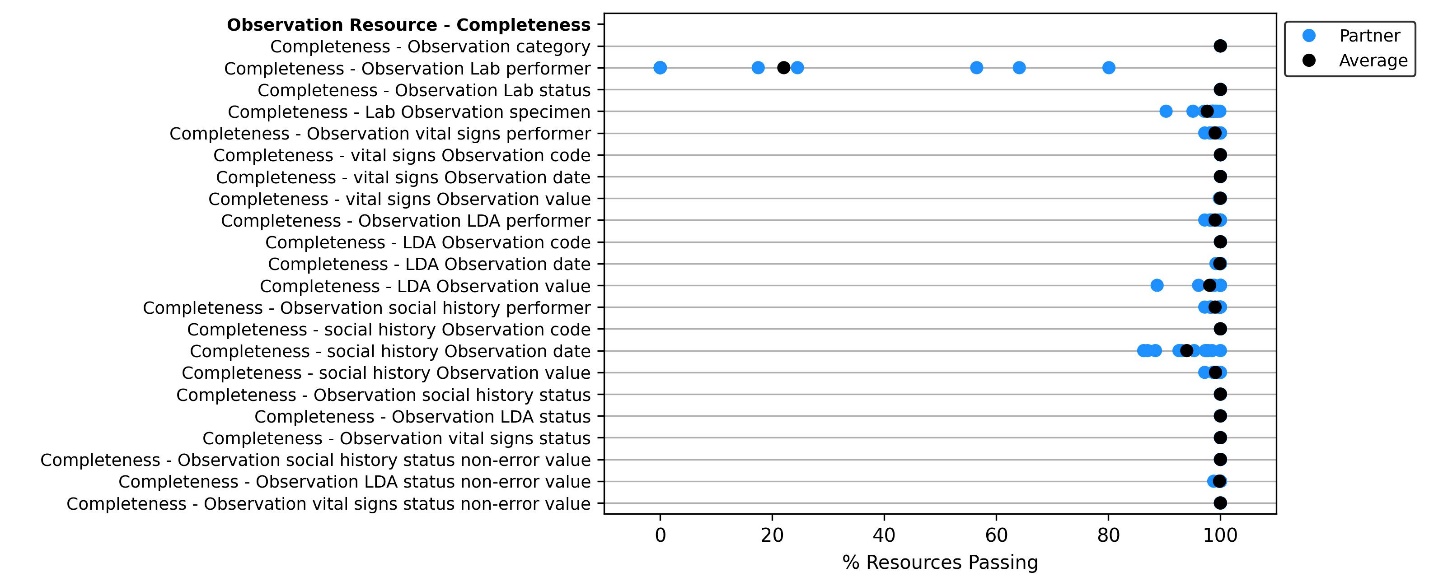
**

Figure X9. Comparison measured by partner average % of resources passing the listed test.

**Figure X10: Data Quality Results for Helpful Data Tests Applicable to Observation Resource (Conformance)**

**
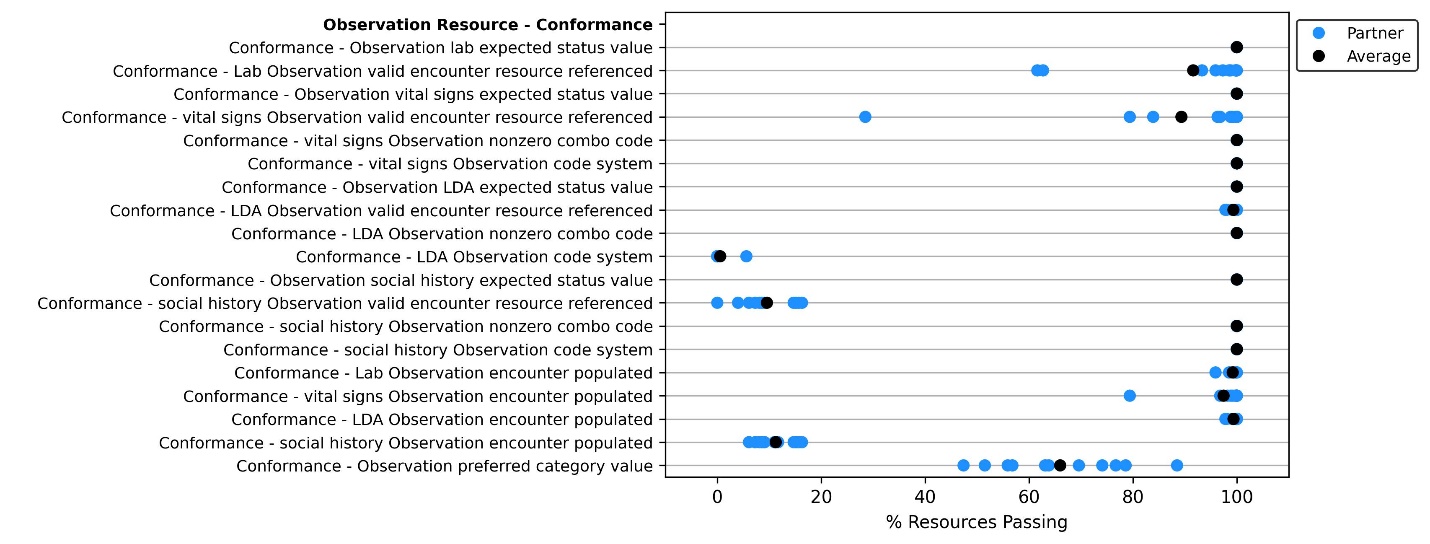
**

Figure X10. Comparison measured by partner average % of resources passing the listed test.

**Figure X11: Data Quality Results for Helpful Data Tests Applicable to Observation Resource (Conformance)**

**
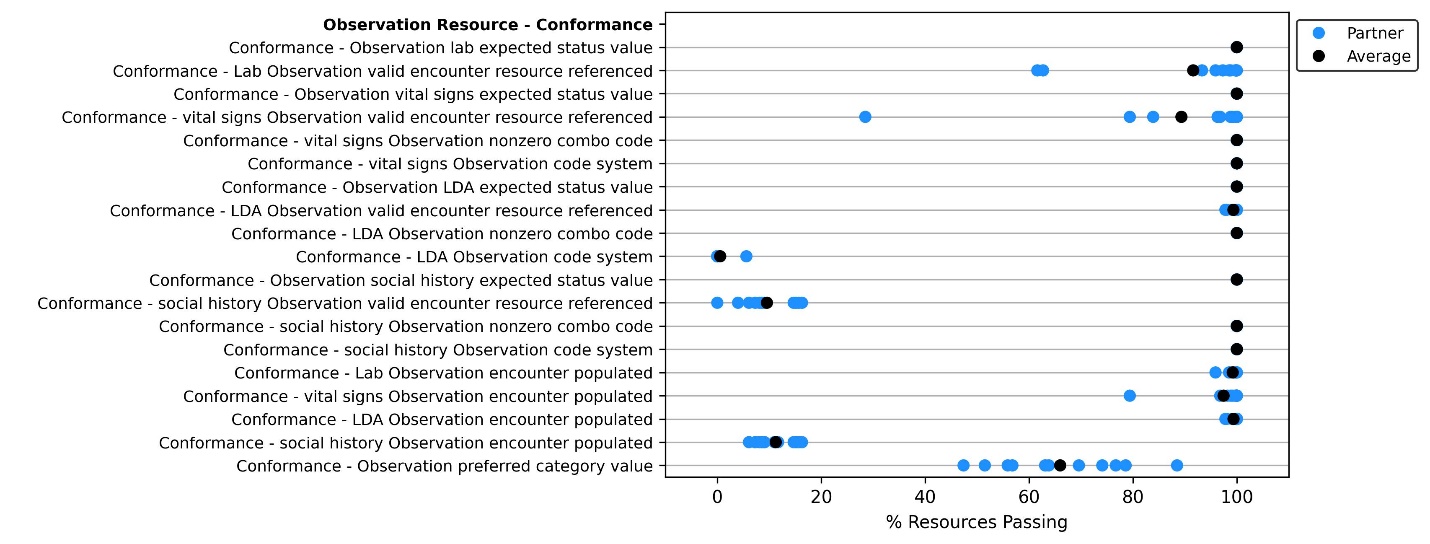
**

Figure X11. Comparison measured by partner average % of resources passing the listed test.

**Figure X12: Data Quality Results for Helpful Data Tests Applicable to Observation Resource (Plausibility) and DiagnosticReport Resource**

**
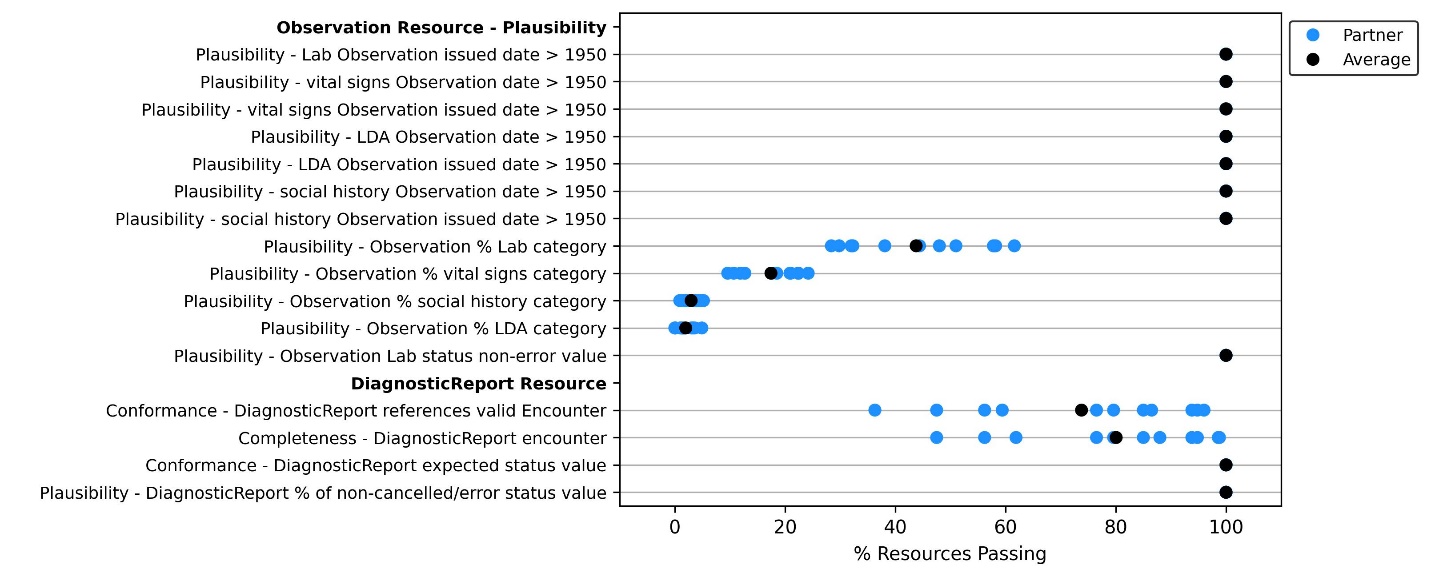
**

Figure X12. Comparison measured by partner average % of resources passing the listed test.

**Figure X13: Data Quality Results for Helpful Data Tests Applicable to Patient and Procedure Resources**

**
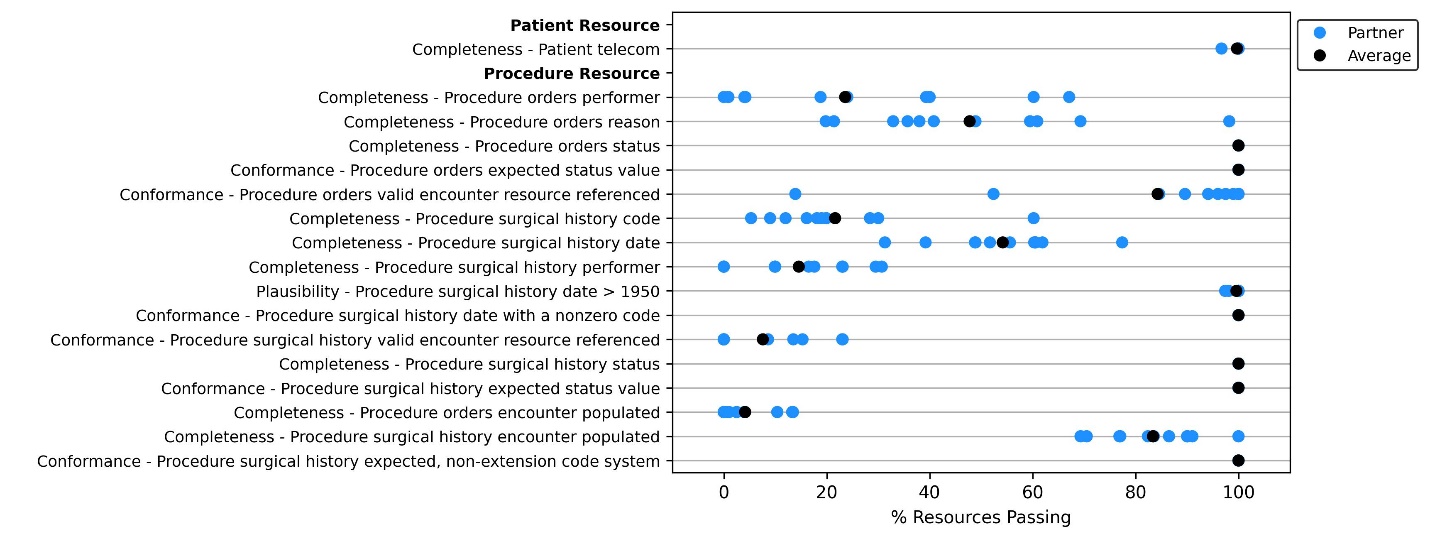
**

Figure X13. Comparison measured by partner average % of resources passing the listed test.

1. HL7. US Core Implementation Guide 2023 [Available from: <https://build.fhir.org/ig/HL7/US-Core/ValueSet-us-core-encounter-type.html>.

2. HL7. HL7 Terminology (THO) 2023 [Available from: <https://terminology.hl7.org/5.1.0/ValueSet-encounter-class.html>.

3. FHIR H. ValueSet Immunization Site 2023 [Available from: <https://build.fhir.org/valueset-immunization-site.html>.
